# Network science inspires novel tree shape statistics

**DOI:** 10.1371/journal.pone.0259877

**Published:** 2021-12-23

**Authors:** Leonid Chindelevitch, Maryam Hayati, Art F. Y. Poon, Caroline Colijn

**Affiliations:** 1 MRC Centre for Global Infectious Disease Analysis, Imperial College London, London, United Kingdom; 2 School of Computing Science, Simon Fraser University, Burnaby, BC, Canada; 3 Department of Pathology & Laboratory Medicine, University of Western Ontario, London, ON, Canada; 4 Department of Mathematics, Simon Fraser University, Burnaby, BC, Canada; Utrecht University, NETHERLANDS

## Abstract

The shape of phylogenetic trees can be used to gain evolutionary insights. A tree’s shape specifies the connectivity of a tree, while its branch lengths reflect either the time or genetic distance between branching events; well-known measures of tree shape include the Colless and Sackin imbalance, which describe the asymmetry of a tree. In other contexts, network science has become an important paradigm for describing structural features of networks and using them to understand complex systems, ranging from protein interactions to social systems. Network science is thus a potential source of many novel ways to characterize tree shape, as trees are also networks. Here, we tailor tools from network science, including diameter, average path length, and betweenness, closeness, and eigenvector centrality, to summarize phylogenetic tree shapes. We thereby propose tree shape summaries that are complementary to both asymmetry and the frequencies of small configurations. These new statistics can be computed in linear time and scale well to describe the shapes of large trees. We apply these statistics, alongside some conventional tree statistics, to phylogenetic trees from three very different viruses (HIV, dengue fever and measles), from the same virus in different epidemiological scenarios (influenza A and HIV) and from simulation models known to produce trees with different shapes. Using mutual information and supervised learning algorithms, we find that the statistics adapted from network science perform as well as or better than conventional statistics. We describe their distributions and prove some basic results about their extreme values in a tree. We conclude that network science-based tree shape summaries are a promising addition to the toolkit of tree shape features. All our shape summaries, as well as functions to select the most discriminating ones for two sets of trees, are freely available as an R package at http://github.com/Leonardini/treeCentrality.

## 1 Introduction

Molecular data describing the evolution, variation and diversity of organisms over time is more widely available than ever before due to rapid improvements in sequencing technology. Using these data to infer the underlying evolutionary process is a key ongoing challenge in many areas of biology. In particular, in infectious disease, it is crucial to understand pathogen adaptation: despite improvements in sanitation and vaccination and the development of antibiotics, infectious pathogens continue to emerge from zoonotic infections and to adapt to human immune responses, vaccines, and antimicrobials. Next-generation sequencing has afforded unprecedented opportunities to generate pathogen genome sequences in a highly scalable manner, and theoretical tools have been developed to interrogate these data, largely through reconstructed phylogenetic trees.

There has been considerable interest over the years in comparing the shapes of phylogenetic trees in order to understand evolutionary processes [1–8]. A tree’s shape specifies its connectivity structure. The lengths of its branches typically reflect either the time or genetic distance between branching events. Following the observation that reconstructed evolutionary trees are more asymmetric than random models predict [9], there have been efforts to summarise tree asymmetry in trees reconstructed from data and relate it to predicted asymmetry in evolutionary and ecological models [4–7, 10–15]. There is also interest in establishing whether taxa from two phylogenies might correspond to each other, for example in the context of parasites and hosts or fossils of different origins [16], and in comparing simulated trees with trees from data in epidemiology, for example using Approximate Bayesian Computation [17–19]. These applications require quantitative tools to compare phylogenetic trees with different taxa, and they require summary features that are informative of the evolution or epidemiology being studied.

Summaries of tree topology have often focused on either asymmetry, or the frequency of various configurations such as cherries or ladders [20]. Well-known measures of asymmetry include the Colless and Sackin imbalance [21, 22]. Asymmetry measures tend to be correlated with each other, and do not fully capture the shape of a tree [16, 23], leading to an interest in exploring other statistics, comparisons tools and metrics for this task [16, 18, 19, 24–27]. Some metric approaches directly find a distance (or similarity) measure between unlabelled phylogenies; others seek an optimal labelling for two unlabelled trees and use metrics for labelled trees, but this is not feasible for large trees.

In one particularly important application of tree shape statistics, namely, Approximate Bayesian Computation, one seeks simulation parameters that produce trees that are similar enough to data-derived trees [28]. This leaves a need for new statistics to quickly provide a good summary of tree topology. Matsen used optimization over binary recursive tree shape statistics to find statistics that distinguish between sets of trees [29]. This set the stage for broadening the set of quantities used to describe trees, and gives rise to natural questions like why a particular recursive statistic separates two groups, whether it only happens to separate those specific trees or acts as a good way to distinguish between trees that are meaningfully similar to those in the two groups. The binary recursive class may furthermore exclude features that capture global information about a tree, such as features derived from its eigenvalues when viewed as a graph, known as spectral features. As the size of datasets and range of applications increases, it is reasonable to assume that inference will be improved by expanding upon the tools available to summarize tree topologies [18, 29]. In applications like Approximate Bayesian Computation, as well as other related attempts at summarizing tree shapes, one does not necessarily seek to relate summaries to evolutionary mechanisms. Rather, the key objective is to distinguish between trees in different categories or scenarios in an efficient way by computing simple statistics.

Network science has become an important paradigm for describing structural (topological) features of networks and using them to understand complex systems, ranging from protein interactions to social systems [30, 31]. Network science is thus a potential source of many novel ways to characterize tree topology. A phylogeny can be interpreted as a simple type of network or graph—specifically, a connected acyclic undirected graph. Thinking of a tree as a graph can lead to a matrix representation (such as the adjacency matrix) in which both rows and columns correspond to its internal and terminal nodes. This makes available the large assortment of techniques for studying the properties of graphs based on their matrix representation [32]. The *spectra* of graph matrices have recently been used to compare tree topologies [26]. However, the use of classical spectra is not likely to uniquely define trees [33, 34], the computation does not scale well with tree size, and describing the relationship between evolutionary or epidemiological parameters and graph spectra may be extremely difficult due to the algebraic rather than combinatorial nature of the latter.

Network science offers many other network features that can be adapted to describe shape. Here, we tailor tools from network science to summarize phylogenetic tree topologies. We thereby develop tree topology summaries that are complementary to both asymmetry and the frequencies of small configurations. These new statistics are fast to compute and will scale well to describe the topologies of large trees. They can additionally be easily adapted to take branch lengths into account. We illustrate how these statistics vary, alongside some conventional tree statistics, over three very different viruses (HIV, dengue fever and measles), and over the same virus in different epidemiological scenarios (influenza A over a 2-year period in the USA, over a 5-year period globally, and over a longer period globally; HIV in three epidemiological contexts, namely a concentrated epidemic in men who have sex with men, a generalised African HIV epidemic in a village setting and HIV-1 from a national-level survey (see Methods)). We also use the statistics to compare simulated trees from different models. Finally, we use classification with and without the network-based statistics to distinguish trees in different settings. We find that the network-science statistics improve the classification performance and are consistently assigned a high importance measure in classification algorithms.

## 2 Methods

### 2.1 Definitions related to graphs

In this subsection we introduce some definitions from graph theory, used below. They are based on the textbooks by Bollobás [35] as well as Godsil and Royle [36].

A *network* or *graph* *G* consists of a finite set of *nodes* *V* and a finite set of *edges* *E* whose elements are unordered pairs of distinct nodes. The edge {*u*, *v*}, denoted *uv* for simplicity, *joins* nodes *u* and *v*, and if *uv* ∈ *E*, the nodes *u* and *v* are *adjacent*, which we denote by *u* ∼ *v*.

A graph is *weighted* if there is a function w:E→R that assigns a *weight* *w*(*uv*) to each edge *uv* ∈ *E*. The weight function extends to all of *V* × *V* by setting *w*(*uv*) = 0 if *u* = *v* or *uv* ∉ *E*.

A *path* *P* in a graph is a sequence of nodes *v*_0_, *v*_1_, …, *v*_*k*_ such that *v*_*i*_ *v*_*i*+ 1_ ∈ *E* for each 0 ≤ *i* ≤ *k* − 1. The nodes *v*_0_ and *v*_*k*_ are the *endvertices*. In the unweighted case, *k* is the *length* of *P*. In the weighted case, the weighted length of the path *P* is measured by the sum of the weights of the edges it contains, i.e. $\sum_{i=0}^{k-1}w\left(v_{i}v_{i+1}\right)$.

In both the unweighted and the weighted cases, the minimum (weighted) length of a path with endvertices *u* and *v* is called the (weighted) *distance* between *u* and *v* and denoted *d*(*u*, *v*). The (weighted) *diameter* of the graph is the largest distance between two distinct nodes.

The *neighborhood* *Γ*(*u*) of a node *u* ∈ *G* is the set of nodes adjacent to it in *G*. The *degree* of *u* is *d*(*u*) = |*Γ*(*u*)|. The *degree sequence* of *G* is the vector **d** = (*d*(*v*_1_)…*d*(*v*_*n*_)) containing the degree of all its nodes in some order. In a weighted graph, the *weighted degree* of *u* is *d*_*w*_(*u*) = ∑_*v*_ *w*(*uv*), and the *weighted degree sequence* is the vector **d**_**w**_ = (*d*_*w*_(*v*_1_)…*d*_*w*_(*v*_*n*_)). The *assortativity coefficient* of *G* is the Pearson correlation coefficient of degree between pairs of adjacent nodes.

Given a numbering of the nodes, *V* = {1, 2, …, *n*}, a graph can be represented by its *adjacency matrix* $A\in R^{n\times n}$, where *A*_*ij*_ = 1 if *ij* ∈ *E* and *A*_*ij*_ = 0 otherwise. If the graph is weighted, its *weighted adjacency matrix* has entries *A*_*ij*_ = *w*(*ij*).

A graph can also be represented by its *Laplacian matrix* $L\in R^{n\times n}$, defined by *L*_*ii*_ = *d*(*i*) for all *i*, *L*_*ij*_ = −1 if *ij* ∈ *E* and *L*_*ij*_ = 0 otherwise. The *weighted Laplacian matrix* has entries *L*_*ii*_ = *d*_*w*_(*i*) for all *i* and *L*_*ij*_ = −*w*(*ij*) if *i* ≠ *j*. Thus, in both cases we have *L* = *D* − *A*, where *D* is the diagonal matrix with the (weighted) degree sequence on the diagonal.

The *normalized Laplacian* [37] is a variant of the Laplacian which is normalized by the degree of each node, and is defined as $L=D^{-1/2}LD^{-1/2}$. Its entries are $L_{ii}=1$ for all *i* and $L_{ij}=-\frac{w(ij)}{\sqrt{d_{i}d_{j}}}$ for *i* ≠ *j* in the weighted case or $L_{ij}=-\frac{1}{\sqrt{d_{i}d_{j}}}$ in the regular case.

In addition, a graph can also be represented by its *distance matrix* $Q\in R^{n\times n}$, with *Q*_*ij*_ = *d*(*i*, *j*), the (weighted or unweighted) graph distance between *i* and *j*, and in particular, *Q*_*ii*_ = 0. As proposed in [26], it is also possible to apply the (regular or normalized) Laplacian transformation to the distance matrix (by treating the distance matrix as a weighted adjacency matrix) to obtain alternative matrix representations of a graph.

Widely studied graph properties fall into two broad classes. The first are *shape properties*, concerned with various connectivity measures of the graph, such as the numbers of distinct paths connecting pairs of nodes and the average or longest distance between a pair of nodes. Some of these properties do not depend on the edge weights of the graph, in which case we refer to them as *topology properties*; we use the term *shape property* as a broad term that includes both topology properties and properties that take edge weights into account. The second are *spectral properties*, concerned with the eigenvalues (and occasionally eigenvectors) of matrices representing the graph, such as the adjacency, Laplacian, and normalized Laplacian.

### 2.2 Definitions related to trees

A tree is a connected acyclic graph. It can be shown that a tree with *n* nodes has *n* − 1 edges, and that there is a unique simple path between any two nodes in a tree. The nodes of degree one are called *tips* (or *leaves*); the remaining nodes are called *internal*. The weight *w*(*e*) of an edge *e* of a tree is also called its *branch length*; we assume that a tree has all positive branch lengths.

A tree *T* is *rooted* if it contains a distinguished node *r*, called the *root*. If *v* is any node other than the root *r* in a rooted tree, the node *u* immediately preceding *v* on the unique simple path from *v* to *r* is called the *parent* of *v* in *T*, and any node *z* on this unique simple path is called an *ancestor* of *v* in *T*. In particular, the root *r* is an ancestor of every node in *T* other than itself. The number of edges on the unique shortest path from a node *v* to the root *r* is called its *depth*.

Conversely, in this situation, *v* is called a *child* of *u* and a *descendant* of *z*. A rooted tree *T* is *binary* if every internal node has exactly two children. In a rooted tree *T*, the set of all descendants of a node *u* including *u* itself forms a *clade*; we say that *u* *subtends* this clade. The *subtree* rooted at *u*, denoted *T*_*u*_, is the clade that *u* subtends together with all the edges of *T* connecting its elements. If a node *u* has two children *v* and *w*, we may arbitrarily refer to them as the left and right child of *u*, and call *T*_*v*_ and *T*_*w*_ the left and right subtree of *u*, respectively.

A tree *T* with root node *r* can be *rerooted* at an internal node *s* ≠ *r*, by simply designating *s* rather than *r* as the root. This changes only the ancestor-descendant relationships, but not the topology of the tree. Note that if *T* is a binary tree with root *r*, *T* rerooted at *s* ≠ *r* will not be binary because *s* will have three children while *r* will have only one child after rerooting. However, this situation is often resolved by specifying a branching order, i.e. creating a new node *t* that is the parent of two children of the new root *s*, and adding an *s* − *t* edge of length 0. An analogous procedure, called “multichotomy resolution”, may be iteratively applied to any other internal nodes with a degree greater than 2 to turn any non-binary tree into a binary tree.

**Fig 1:**
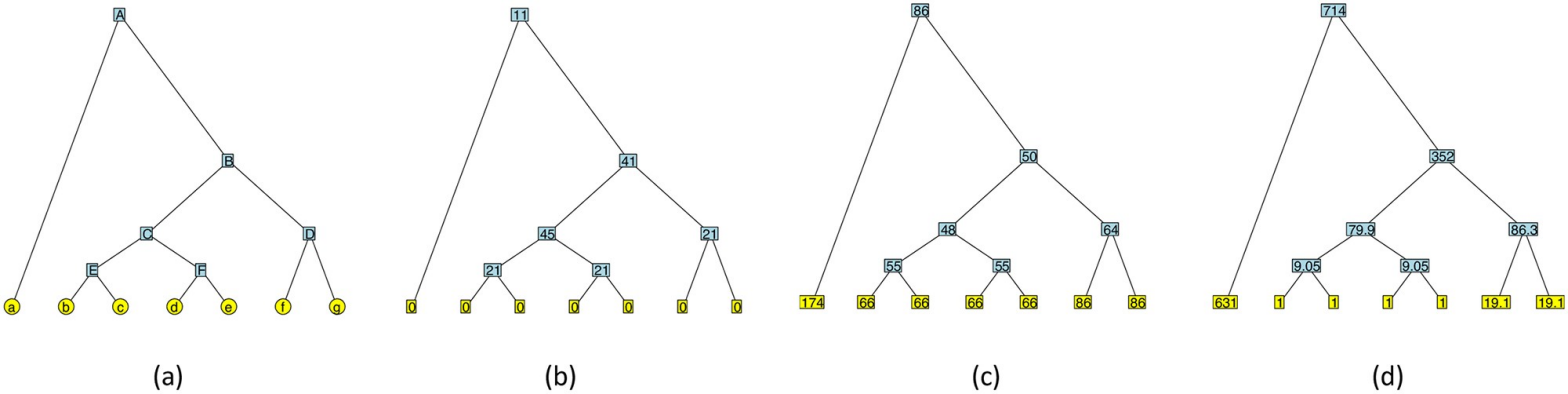
(a) An example tree. In the epidemiological context, tips (*a* − *g*) would correspond to pathogen sequences and internal nodes (*A* − *F*) to their inferred common ancestors. Node *D* subtends a “cherry” configuration, and node *C* subtends two cherries (a “double cherry”). The heights of the internal nodes are 1 (*E*, *F*), 2 (*C*, *D*), 4 (*B*) and 8 (*A*), so the diameter is 16 and the Wiener index is 484, for a mean path length of 6.21. (b) Same tree with betweenness centrality values at each node (note that branch lengths do not change them). The tree has betwenness centrality 45. (c) Same tree with farness (reciprocal of closeness centrality) values at each node. The tree has closeness centrality 1/48. (d) Same tree with eigenvector centrality values (scaled to have a minimum of 1) at each node, rounded to 3 significant figures. Here, the leading eigenvalue is λ = 9.05. By our definition, the tree has eigenvector centrality 714/1023 = 0.698 (here, 1023^2^ is the sum of the squared values).

A molecular *phylogeny*, or *phylogenetic tree*, is a binary rooted tree whose tips correspond to genetic or genomic sequences, and whose internal nodes represent their inferred common ancestors. A phylogeny therefore represents the ancestral relationships among a set of genomes. The shape of a phylogeny tells the story of an evolutionary history going back through time to the most recent common ancestor at the root of the tree (see Fig 1(a)).

### 2.3 Data and simulations

### 2.3.1 HIV/Dengue/Measles

We obtained Newick tree strings corresponding to phylogenies inferred from human and zoonotic RNA viruses from a previous study. Specifically, we retrieved tree strings reconstructed from genetic sequences of HIV-1 subtype B, Measles virus, and Dengue virus serotype 4. The HIV sequence data (corresponding to the gene encoding the Nef protein) were obtained from the LANL HIV Sequence database [38], through the web site at http://www.hiv.lanl.gov, and screened for recombinants using the SCUEAL algorithm [39]. The remaining virus sequences were obtained from GenBank [40].

Phylogenies were reconstructed from random samples of 100 sequences by maximum likelihood using RAxML [41] under a general time-reversible model of nucleotide substitution and rate variation among sites approximated by the GTRCAT model. HIV-1 subtype B phylogenies were rooted using a subtype D sequence as an outgroup. Dengue virus serotype 4 phylogenies were rooted on an outgroup sequence isolated in the Philippines in 1956. Finally, measles virus phylogenies were rooted using a genotype D6 sequence as the outgroup. The GenBank [40] accession numbers for all outgroups can be found S1 Table in S1 File.

### 2.3.2 HIV in three settings

We obtained HIV-1 sequence data from three published studies. The Wolf et al. [42] data set corresponds to samples from a concentrated epidemic of HIV-1 subtype B in populations of predominantly men who have sex with men in Seattle, USA. The Novitsky et al. [43] data set corresponds to samples from a generalized epidemic of HIV-1 subtype C infections in Mochudi, Botswana, a village with an estimated HIV-1 prevalence of about 20% in the adult population. Similarly, the Hunt et al. [44] data set represents samples from a national survey of the generalized epidemic of HIV-1 subtype C in South Africa. Thus, these studies represent a range of geographic scales and epidemiological contexts.

### 2.3.3 Influenza in three settings

We compared the topologies of a single virus (human influenza A) sampled to reflect different epidemiology: (1) samples over a five-year period; (2) global samples over a 12-year period and (3) samples from 2012–2013 from the USA only. We downloaded full-length hemagglutinin (HA) sequences of human H3N2 flu from NCBI and aligned sequences from each group with MAFFT [45]. For each sample, we chose 120 sequences uniformly at random from the alignment, and inferred a tree with these sequences as tips using IQtree [46] with the pll (phylogenetic likelihood library) option [47] and the GTR+ G model. Using the date information from NCBI, we rooted the trees using the root to tip (*rtt*) function in the ape package [48] in R [49].

### 2.3.4 Simulated tree models

We simulated trees from four random processes: a Yule process (pure birth trees), a “biased” model of Kirkpatric and Slatkin [5] in which speciation rates are unevenly assigned to a node’s descendants with a bias (here 0.3), and two constant rate birth-death processes, with the basic reproduction number (mean of the offspring distribution) equal to 1.5 and 3. We created sets of 100 trees with 100 tips and separately with 300 tips. The apTreeshape package [50] was used to simulate the Yule and biased models; tree shapes were converted to phylogenetic trees using the as.phylo function. We used the TreeSim package [51] for the birth-death models. Because in sim.bd.taxa (in TreeSim) the simulations are conditioned on having a fixed number of extant tips, we created trees with 300 or 600 extant tips and randomly pruned taxa to leave a tree of 100 or 300 tips, modelling partial sampling over time.

As some scenarios can be distinguished simply by comparing the branch lengths of the corresponding trees, we normalized the time scales so that each of our trees has a mean branch length of 1. This ensures that any differences we observe between the summary statistics in different classes are not simply due to scaling. We did not, however, modify the variances of the branch length distribution, as those may contain some of the signal picked up by summary statistics.

In total, there are 5 scenarios in which we compare trees: HIV/Dengue/Measles (HDM), influenza (2-year USA, 5-year global, 12-year global), HIV contexts (labeled WNH after the first author names of the corresponding publications), simulated trees with 100 tips (’Simulated’) and simulated trees with 300 tips (’Simulated300’). Within each set, we performed classification with generalized linear models and random forests, using the tree shape statistics as features. We computed a measure of each feature’s importance for each classification. All reconstructed trees, including accession numbers (in the tip labels), have been deposited to http://github.com/Leonardini/treeCentralityData.

### 2.4 Shape features

We computed a range of topological and spectral summary features of the viral phylogenies (see Table 1 for the definitions and references for each one). Our focus here is on some of the novel tree topology summary statistics, but we also include a number of standard statistics for comparison. All input trees were binary and rooted, and all branch lengths were non-negative, although many of the trees had zero-length branches. All comparisons involved trees on the same number of tips. In order to enable comparisons between trees with different numbers of tips, we also provide, in the S1 File, some partial results on the order of magnitude of the maximum and minimum possible values of each newly introduced statistic over all trees of a certain size.

**Table 1:** Summary measures for phylogenetic trees. Here, *n*_*i*_ is the number of nodes at depth *i*.

Name	Description	Short form	Ref.
Numbers of small configurations			
Cherry number	# of nodes with 2 tip children	cherries	[52]
Pitchforks	# of nodes with 3 tip descendants	pitchforks	[20]
Double cherries	# of nodes with 2 cherry children	doubcherries	new
4-caterpillar	# of caterpillars with 4 tips	fourprong	[20]
Clades of size *x*	# of nodes with *x* tip descendants	num*x*	[20]
Tree-wide summaries			
Colless imbalance	Colless imbalance	colless	[21]
Sackin imbalance	Mean path length from tip to root	sackin	[22]
Maximum height	Max # of steps from the root	maxheight	[53]
Maximum width	Max # of nodes at the same depth	maxwidth	[53]
Stairs	Proportion of imbalanced subtrees	stairs	[54]
Max difference in widths	max_*i*_(*n*_*i*+1_ − *n*_*i*_)	delW	[53]
Node properties from network science			
Betweenness centrality	# of shortest paths through node	between	[55]
Weighted betweenness	as above, but with weighted edges	betweenW	[56]
Closeness centrality	total distance to all other nodes	closeness	[55]
Weighted closeness	as above, but with weighted edges	closenessW	[56]
Eigenvector centrality	value in Perron-Frobenius vector	eigen	[55]
Weighted eigenvector	as above, but with weighted edges	eigenW	[56]
Summaries from network science			
Diameter	largest distance between 2 nodes	diameter	[35]
Mean pairwise distance	average distance between 2 nodes	meanpath	[57]
Spectral properties			
Min adjacency	min adjacency matrix eigenvalue >0	minAdj	[36]
Max adjacency	max adjacency matrix eigenvalue	maxAdj	[36]
Min Laplacian	min Laplacian matrix eigenvalue >0	minLap	[36]
Max Laplacian	max Laplacian matrix eigenvalue	maxLap	[36]
Distance Laplacian spectral properties			
Max eigenvalue	largest eigenvalue in the spectrum	dLapLambdaMax	[26]
Max density	location of largest spectral density	dLapDensityMax	[26]
Asymmetry	skewness of the spectral density	dLapAsymmetry	[26]
Kurtosis	peakedness of the spectral density	dLapKurtosis	[26]

For the node properties derived from network science, we focus our discussion on the maximum value of each type of centrality a node can have within a tree, but using other derived statistics (the minimum, mean, median or variance) could also have been an option. However, one advantage of using the maximum (the minimum tends to be less informative as it is 0 for many of the measures we consider) instead of the other possible options is that it takes on integer values for unweighted trees, which is not usually the case of the mean, the variance, or the median when there is an even number of nodes. For the spectral properties, which also produce a value for each node, we focus on the maximum value as well as the minimum strictly positive value. Lastly, for the distance Laplacian spectral properties we use the four derived statistics proposed by Lewitus and Morlon [26].

#### 2.4.1 Tree topology summaries based on network science

Network science, broadly defined as the study of complex networks, has produced a number of tools that have been applied in a variety of contexts [30, 31], including degree sequence, degree assortativity, density, diameter, and a number of node centrality measures. Only some of these apply naturally and informatively to phylogenies. For instance, the degree sequence, the degree centrality, the density and the clustering coefficients do not vary between phylogenetic trees on *n* tips, while their degree assortativity can take on one of two values which are close to one another for large *n* (see section section 4 in S1 File for details).

We now discuss those network science-inspired features that are informative for phylogenetic trees.

#### 2.4.2 Diameter, average shortest path and Wiener index

The diameter (the maximum length of a shortest path) is a useful summary statistic. For general trees with *N* nodes, it can be calculated in linear time using a “folklore” dynamic programming algorithm, and its value can vary between 2 for the star and *N* − 1 for the path of length *N*. For phylogenetic trees with *N* = 2*n* − 1 nodes, the range is from ≈ 2log_2_(*n*) to *n*. The exact maximum and minimum values, as well as the trees attaining them, and the distribution for *N* = 45(*n* = 23), are described in S10 Fig in S1 File.

The average shortest path length of a tree may also be informative. Unlike for general graphs, it can be calculated in linear time in a tree with *N* nodes using dynamic programming [57]. The sum of all shortest path lengths between pairs of nodes in a tree is known as the tree’s Wiener index, and equals (2*n* − 1)(*n* − 1) times the average shortest path length. The Wiener index for general trees with *N* nodes is always contained between (*N* − 1)^2^ and (N+13), values which are attained by the star and the path, respectively [58]. For phylogenetic trees, the range of the Wiener index is substantially narrower; its distribution for *N* = 45(*n* = 23) is described in S11 Fig in S1 File. Note that both the diameter and Wiener index generalize naturally to trees with arbitrary positive branch lengths.

Based on their distributions for small phylogenetic trees, shown in S12 Fig in S1 File we can think of trees that have a diameter or a Wiener index much smaller than the mean as being similar to complete binary trees, and of those that have a diameter or a Wiener index much larger than the mean as being similar to caterpillars or double caterpillars, respectively. This is not too different from the corresponding situation for imbalance statistics such as Colless or Sackin index. However, the situation changes dramatically when we look at node centrality measures defined in network science. A number of such centrality measures exist that can be defined for each node of a graph.

In particular, betweenness centrality, closeness centrality and eigenvector centrality are quantities derived from network science that can be computed in linear time for a tree with *N* nodes and can capture aspects of tree topology not captured by mere asymmetry.

#### 2.4.3 Betweenness centrality

Betweenness centrality associates to each node *v* in a graph the number of pairs *u*, *w* ∈ *V* − {*v*} such that the shortest *u* − *w* path passes through *v*; in other words,

$$\begin{array}{c} C_{B}\left(v\right)≔|\{(u,w)\in V-\{v\}|d(u,w)=d(u,v)+d(v,w)\}|. \end{array}$$

Betweenness centrality can be normalized by the number (N-12) of all pairs *u*, *w* ∈ *V* − {*v*}, but we choose not to use this normalization here. In a tree *T*, there is a unique shortest path between every pair of nodes, and the shortest *u* − *w* path passes through *v* if and only if *u* and *w* are in clades subtended by different children of *v* when *T* is rerooted at *v*. Hence, the betweenness centrality of an internal node *v* is simply *Π*_*i* < *j*_ *n*_*i*_ *n*_*j*_, where *n*_1_, …, *n*_*k*_ are the sizes of the clades subtended by the *k* children of *v* when *T* is rerooted at *v*, and *k* is the degree of *v* in *T*. This implies that the betweenness centrality of all the nodes in a tree can be computed in linear time, a result that, while not surprising, does not seem to have been mentioned in the literature until now, although an algorithm to perform this computation in a distributed fashion has been published [59]. This contrasts with the situation for a general graph, where the best algorithm takes *O*(*NM*) time, where *M* is the number of edges, making it quadratic time for trees [60].

In a phylogenetic tree, the degree is 1 for a tip, 2 for the root and 3 for internal nodes, so the betweenness centrality is respectively 0, *n*_1_ *n*_2_ or *n*_0_ *n*_1_+ *n*_0_ *n*_2_+ *n*_1_ *n*_2_ in those cases, where *n*_1_ and *n*_2_ are the sizes of the left and right subtrees of the node and *n*_0_ is the number of nodes outside the subtree rooted at this node. This can easily seen to be maximal when *n*_0_ = *n*_1_ = *n*_2_, a situation that does not occur in every tree, but is only possible in those with *N* ≡ *n* ≡ 1 mod 3. The betweenness centralities are shown for all the nodes of an example tree in Fig 1(b). Trees with high maximum betweenness centrality are those which have a node whose left subtree, right subtree, and “outside subtree” (what remains of the tree after removing the subtree rooted at the node), are all close in size—a kind of three-way symmetry, as opposed to the two-way (left-right) symmetry measured by classical statistics. The distribution of this quantity, maximum betweenness centrality, for *N* = 45(*n* = 23), is described in S13 Fig in S1 File. We also note that the betweenness centrality values of a tree do not change if the tree has variable branch lengths, since each pair of nodes is connected by a unique shortest path. However, this observation is not true for more complicated graphs.

#### 2.4.4 Closeness centrality

Closeness centrality associates to each node *v* in a graph the inverse of the sum of its distances to all the other nodes in the graph. In other words,

$$\begin{array}{c} C_{C}\left(v\right)≔\frac{1}{\sum_{u}d\left(u,v\right)}. \end{array}$$

The definition means that closeness centrality is inversely proportional to *farness*, the sum of distances from a node to all the other nodes in the graph; hence, it will be small for centrally located nodes and large for remote ones. While this quantity generally requires at least *O*(*NM*) time to be computed for a graph with *N* nodes and *M* edges [61], this can be reduced to linear time for a tree, an observation that does not seem to have been published previously, although a distributed algorithm for performing this computation has been proposed [62].

Indeed, if we consider an internal node *u* with a child *v*, we have *d*(*v*, *x*) = *d*(*u*, *x*) − 1 for every node *x* in the clade of *T* that *v* subtends, and *d*(*v*, *y*) = *d*(*u*, *y*) + 1 for every node *y* outside this clade. Hence, by computing the height (distance to the root) of each node and the sizes of the left and right subtrees of each node in a bottom-up traversal of the tree, we can also find the closeness centrality in linear time for all the nodes in the tree. This is illustrated on our example tree in Fig 1(c). It can also be shown—as we do in Section 7 in S1 File in the S1 File—that the largest value of closeness centrality in a phylogenetic tree is achieved by its unique centroid (the node *v* such that, if the tree is rerooted at it, no child of *v* subtends a clade with more than half of all the nodes), while the smallest value is achieved by one of the tips (in fact, this result also holds for trees with arbitrary positive branch lengths). The distribution of this quantity, maximum closeness centrality, for *N* = 45(*n* = 23), is described in S14 Fig in S1 File.

#### 2.4.5 Eigenvector centrality

Eigenvector centrality associates to each node *v* in a connected weighted graph the positive value *e*(*v*) such that

$$\begin{array}{c} \sum_{u∼v}w\left(uv\right)e\left(u\right)=\lambda e\left(v\right) \end{array}$$

holds for all nodes simultaneously with the largest possible λ. In other words, $\vec{e}$ is the Perron-Frobenius eigenvector of the graph’s adjacency matrix. Since this vector is only defined up to a constant, we use the default normalization, which takes it to be a unit vector in the Euclidean norm. We show an alternative normalization, which makes the minimum entry 1, in Fig 1(d).

Eigenvector centrality is closely related to the adjacency matrix-based spectral methods we discuss below. Since the adjacency matrix of a phylogenetic tree with *n* tips has exactly 4*n* − 4 non-zero entries, it follows that eigenvector centrality can also be computed to a fixed precision *ϵ* in linear time, with a constant proportional to log(1/*ϵ*). It can also be shown—as we do in Section 7 in S1 File—that the largest value of eigenvector centrality in a phylogenetic tree is achieved by an internal node, though it will not necessarily be the root (this result holds for trees with arbitrary positive branch lengths). The distribution of this quantity, maximum eigenvector centrality, for unweighted (unit branch length) phylogenetic trees with *N* = 45(*n* = 23), is shown in S15 Fig in S1 File.

#### 2.4.6 Spectral properties of trees

Spectral properties are properties of the eigenvalues of matrices associated with graphs, such as the adjacency, Laplacian or normalized Laplacian matrices [36]. Since spectral methods are a key tool of algebraic graph theory, it is natural to ask whether they can capture important features of pathogen phylogenies. Some of the eigenvalues are interpretable in a limited number of cases [36]. For instance, the eigenvalues of the Laplacian of a graph reveal the minimal energy of its balanced orthogonal representation in $R^{m}$. The second smallest eigenvalue provides a bound on the *conductance* of the graph, the minimum ratio of the number of edges crossing a cut to the size of the cut. The eigenvalues of the normalized Laplacian also provide information on the conductance of a graph as well as its diameter, number of bipartite components and properties of random walks defined on it [37].

Since trees are bipartite graphs, the spectra of their adjacency matrices are symmetric around 0 [36] and the spectra of their normalized Laplacians are symmetric around 1 [37]. The full set of eigenvalues of a tree’s adjacency matrix reveals the number of *matchings* (node-disjoint collections of edges); namely, the characteristic polynomial, which has these eigenvalues as its roots, has coefficients whose absolute values equal the number of matchings of each size [34].

#### 2.4.7 Spectral properties derived from the distance Laplacian matrix

In a recent paper, Lewitus and Morlon [26] used the spectra of the distance Laplacian matrix, obtained by subtracting the distance matrix from a diagonal matrix formed by its row sums, to characterize and compare trees. They found promising results and tentatively stated that these spectra were likely to distinguish trees from one another. In Section 8 in S1 File, on the negative side, we exhibit a pair of trees on *n* = 3 tips with different branch lengths that nevertheless have identical distance Laplacian spectra. On the positive side, we verified that no pair of different phylogenetic trees with at most 32 tips and unit branch lengths has this property; however, we suspect that, as has been shown for most other spectra [34], the fraction of trees on *n* tips uniquely defined by their distance Laplacian spectra tends to 0 for large *n*.

We use the four summary statistics proposed by the authors [26]—namely, the maximum eigenvalue, and the asymmetry, kurtosis, and maximum density of the eigenvalue distribution (obtained via smoothing with a Gaussian kernel), as implemented in the RPANDA [63] package in R [49]. We note that, unlike the rest of the statistics in this paper, these ones require a number of operations proportional to *n*^3^, where *n* is the number of tips, and thus take substantially longer to compute.

## 3 Results

### 3.1 Tree topology differentiates viruses and epidemiological scenarios

**Fig 2:**
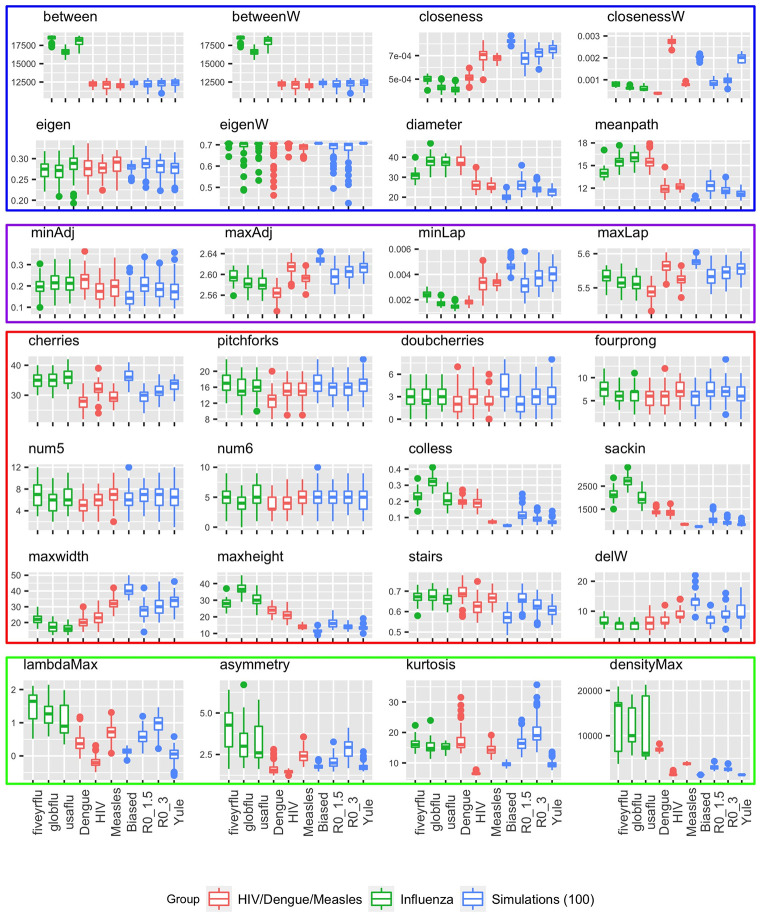
Tree summaries for the HIV/Dengue/Measles, influenza and simulations of size 100.

**Fig 3:**
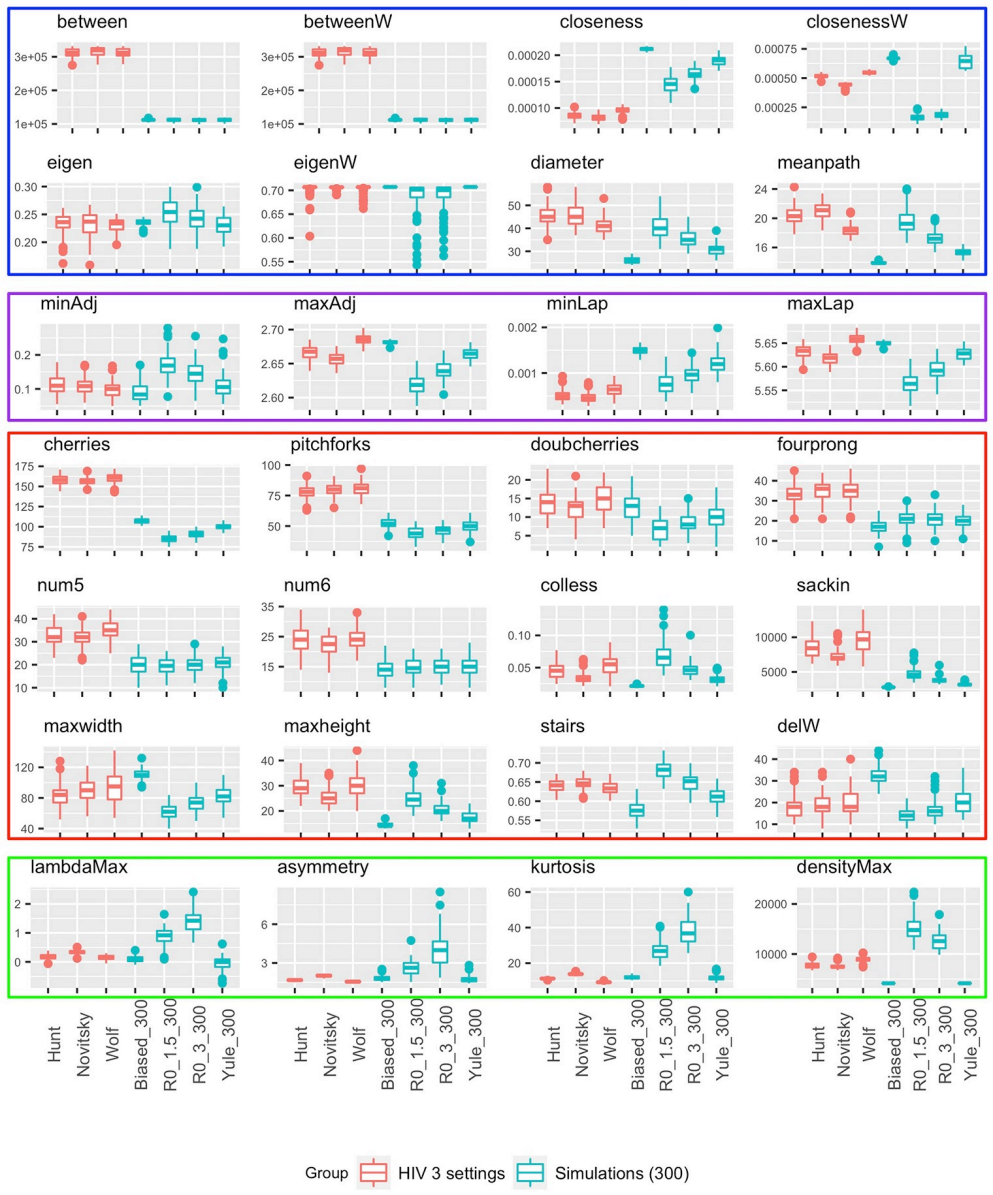
Tree summaries for HIV in three settings and simulations of size 300.

We find that tree topology carries considerable information and differs both between viruses and between different epidemiological scenarios for the same virus, on real as well as simulated data. While degree sequence, clustering coefficients and other measures based in network science are not informative for binary trees, a number of non-standard topological features differ. Furthermore, there are several features that distinguish well between groups of trees with the same overall level of asymmetry, highlighting the need to move beyond asymmetry when using trees to infer evolutionary and epidemiological parameters or to test hypotheses [64]. Figs 2 and 3 illustrate the distributions of all tree statistics considered in this paper. In addition, we perform a (two-sided) Mann-Whitney U test, also known as a Wilcoxon rank-sum test [65], between each pair of scenarios or viruses in a given group, for every statistic we consider, and report the *p*-values in S2 and S3 in S1 File. We then apply a Bonferroni correction [66] to account for the multiple hypotheses being tested, and highlight in bold those values which remain significant at the *α* = 0.05 level after this correction.

Most of the statistics do not vary much between the three viruses in Fig 2 (red boxplots). Distinguishing the topologies in these groups of trees requires tools going beyond the traditional symmetry or imbalance metrics; in this case, the only ones that produce statistically significant differences between all three pairs of viruses are the number of cherries, maximum height, maximum width, and the proportion of imbalanced subtrees (stairs); all of these capture differences that are not apparent in the imbalance. In contrast, while two of the spectral statistics (maximum adjacency and maximum Laplacian eigenvalues) show statistically significant differences among the groups, the vertical scale is very small, and these differences are a small percentage of the overall value (2% for the maximum adjacency and 1% for the maximum Laplacian). Furthermore, one of the network statistics—namely, the weighted closeness centrality—also distinguishes the three viruses, as do three of the four statistics based on the distance Laplacian.

**Fig 4:**
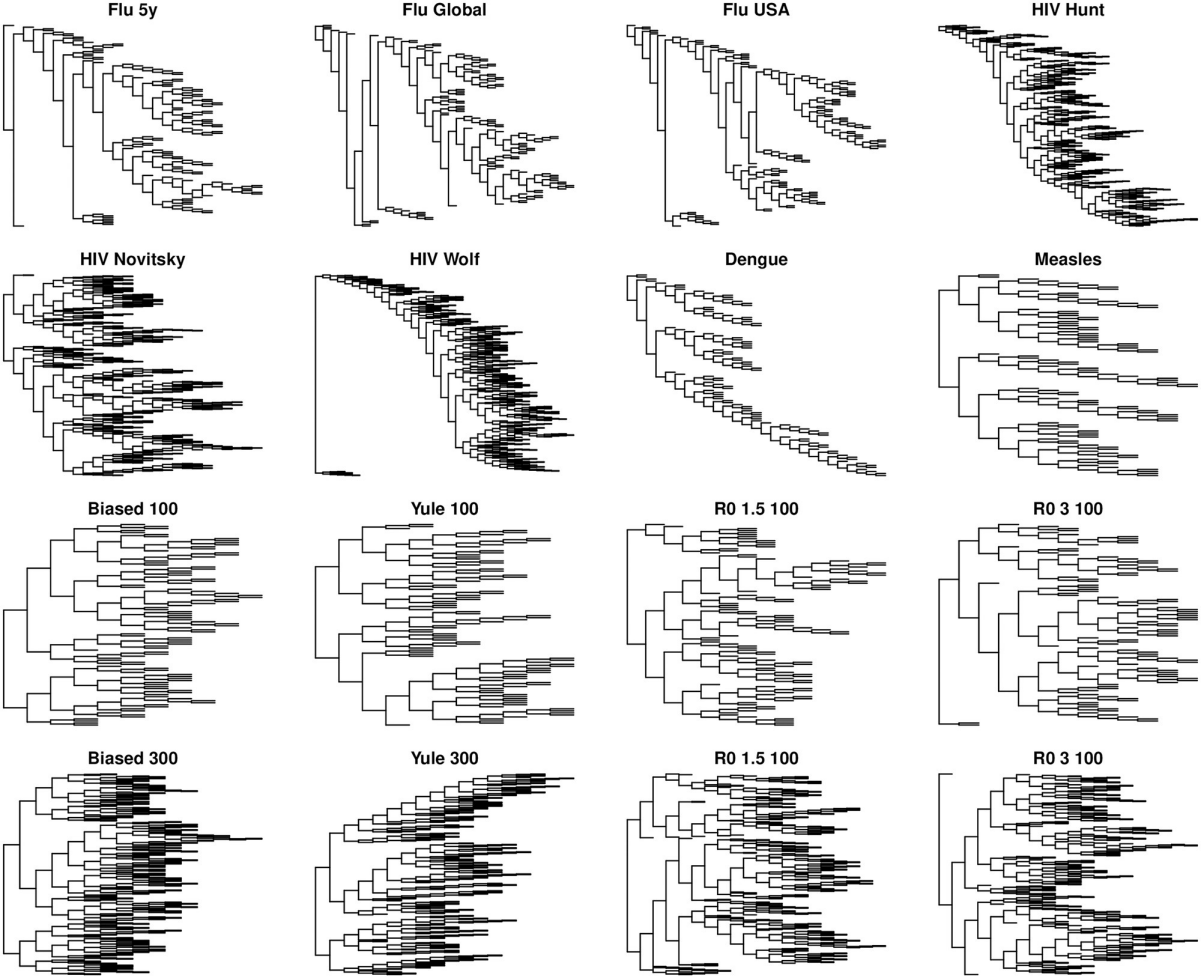
A randomly sampled tree from each scenario (except HIV in the 3-virus comparison because HIV is represented in three other trees). To allow for focus on tree shape rather than on branch lengths, trees have been visualized with branch lengths set to 1.

The green boxplots in Fig 2 show the tree summaries for influenza in three scenarios. It is well-known that global influenza patterns give rise to highly imbalanced trees, an observation which in part motivated the growing field of phylodynamics [64]. In our data, the global five-year and (2-year) USA flu trees have similar, lower, levels of asymmetry. The only statistics which are able to differentiate all three groups at a statistically significant level after multiple testing correction are betweenness centrality and the maximum Laplacian eigenvalue. As with the three viruses, the differences in the latter are not pronounced, and are very small in relative magnitude. In addition, although the LM spectral statistics visually appear to differ between the types of flu, none of these differences is statistically significant. Fig 4 shows a random tree from each group, and differences between the shapes of the flu trees are not immediately apparent by eye.

The blue boxplots in Fig 2 show the summaries for small trees (100 tips) in the four simulated settings. This time, only the closeness centrality, the diameter, and the mean path, as well as the number of cherries, the Sackin and Colless imbalances, the maximum height, the stairs statistic and the lambdaMax statistic based on the distance Laplacian spectrum, are able to differentiate every pair of these scenarios in a statistically significant way.

In contrast, the cyan boxplots in Fig 3 show that large trees in the same simulated settings can be discriminated by all of these statistics, but also by several other ones, including the adjacency and Laplacian eigenvalues as well as the maximum width and the maximum difference in width (delW). This suggests that it is strictly easier to discriminate larger trees than it is to discriminate smaller trees.

Lastly, the three epidemiological scenarios (concentrated epidemic, generalized epidemic in a village, and generalized epidemic in a country) for HIV, shown in the red boxplots in Fig 3, appear to be quite difficult to distinguish. Only the weighted and unweighted closeness, as well as the Sackin and Colless imbalance, the maximum eigenvalues of the adjacency and the Laplacian, and the asymmetry and kurtosis of the distance Laplacian spectrum, are able to do so in a statistically significant manner.

**Fig 5:**
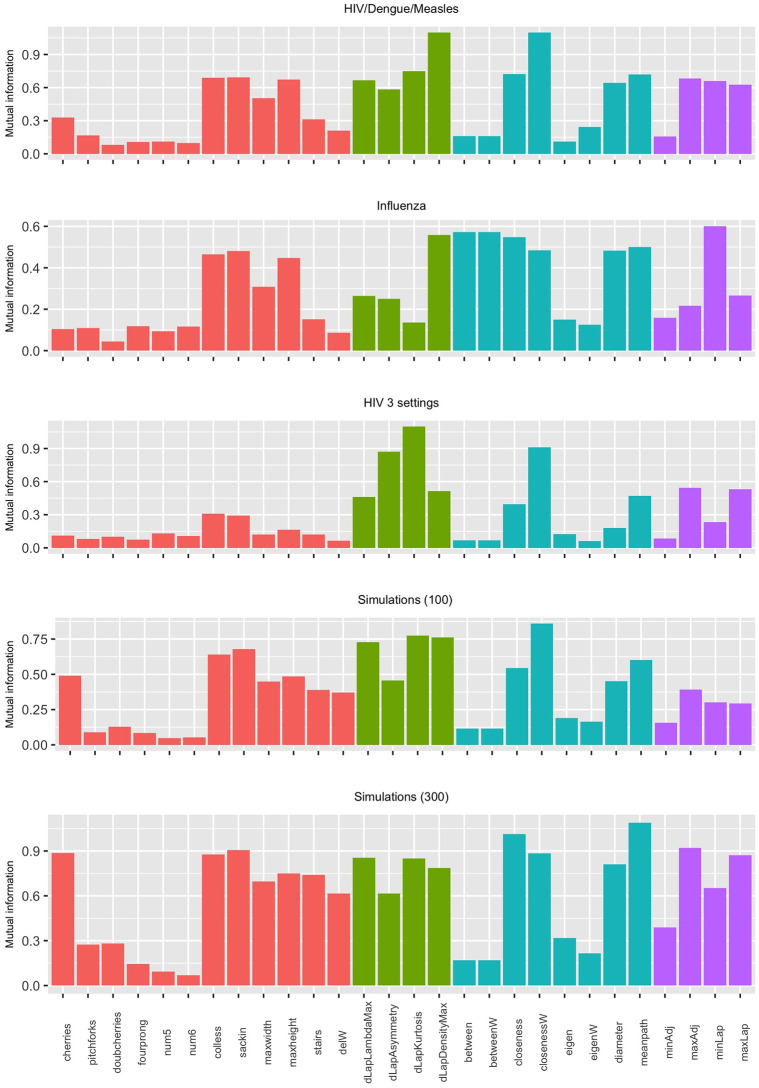
Mutual information between tree summaries and the virus type or epidemiological scenario. Labels are as in Table 1, and the colour indicates the type of statistic as per Table 1, i.e. red: basic statistics; green: distance Laplacian spectrum statistics; blue: network science-based statistics; purple: spectral statistics.

Fig 5 shows the mutual information between the virus or epidemiological scenario and the value of each statistic, for the 5 settings we tested. We have computed these values by discretizing the range of each tree shape statistic into 20 equally-sized bins. The first panel shows that the highest mutual information with the virus (HIV, Dengue or Measles) is obtained by a network science-based statistic in Fig 5, namely, the weighted closeness, with the densityMax coming close second, while the second panel shows that the highest mutual information with the type of flu (global, US or 5-year) is obtained by the minimum Laplacian eigenvalue, with the betweenness (which is the same whether it is weighted or unweighted) and densityMax coming close behind. In the remaining three scenarios (third, fourth and fifth panels), closeness centrality is the only statistic consistently in the top 3 highest mutual information with the model category or scenario.

### 3.2 The addition of network statistics improves classification

In some scenarios, the trees are markedly different and only a few features are necessary to distinguish them. For example, in the influenza scenario it is clear from Fig 3 that the Colless, Sackin, betweenness and maximum heights statistics all distinguish between global influenza and the other two groups, whereas the closeness measures, the diameter, the mean path and the maximum width distinguish the five year trees from the other two groups. Asymmetry measures alone do not distinguish the three groups.

**Fig 6:**
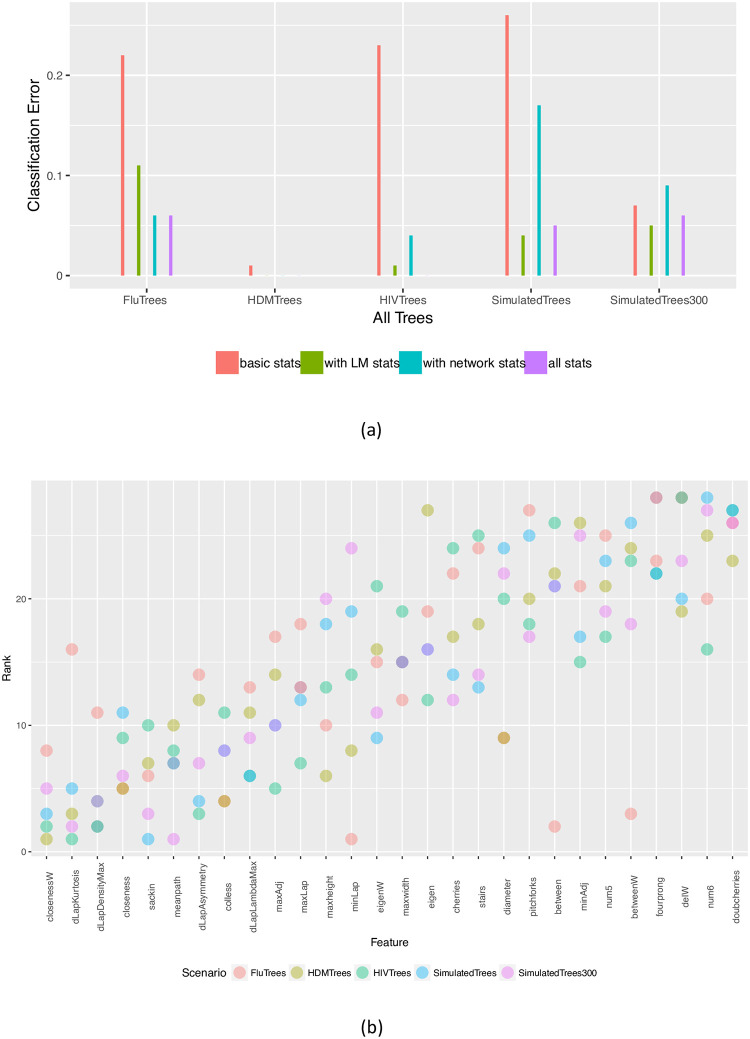
(a) Accuracy of classification with and without network science features, as well as with and without the distance Laplacian spectral features. (b) Feature importance in multi-class classification across all scenarios, ordered by the median. Here the importance for each feature is computed based on the mean decrease in node impurity (we use the *varImp* function in *R*, which uses the Gini index as an impurity measure). Each point is the rank of the corresponding feature in one of the classification tasks. Low ranks correspond to the most important features (i.e. the top-ranked feature has rank 1, and so on).

We use a generalised linear model and two flavours of random forests to classify trees within each scenario. For example, we classified trees as HIV, Dengue or Measles; we classified influenza trees as five-year, global, and USA; we classified simulated trees as biased, Yule, *R*_0_ = 1.5 or 3; and we classified the HIV trees by epidemiological scenario. In each classification task, we randomly selected 75% of the trees (75 trees from each group) for training and used the remaining 25 trees from each group for testing, and computed the classification error of the predictor on the testing set. We report the overall classification error with and without the features based on network science, as well as with and without the features based on the distance Laplacian spectrum (abbreviated as “LM” statistics after the authors Lewitus and Morlon). The results are shown in Fig 6(a). It is clear that the standard (“basic”) tree shape statistics are not able to get close to perfect classification on any of the datasets except for the (relatively simple) task of distinguishing three different viruses. The addition of the costly LM statistics improves the performance, but so does the addition of the easily computable new network science-based statistics we introduce in this paper, with comparable gains in performance relative to the baseline (a larger improvement on the flu scenarios, but a smaller one on the HIV scenarios and on simulated data). Interestingly, the addition of network statistics actually increases the error relative to the baseline on the large simulated trees, an effect that is likely due to the random nature of the classifier.

Both random forests and the GLM regression additionally provide estimates of the importance of each feature. For the random forest classifier, the prediction accuracy on the out-of-bag portion of the data is recorded for each tree, and then the same is done after randomly permuting each predictor variable. The differences between the two accuracies are then averaged over all trees, and normalized by the standard error. For the GLM regression, the absolute value of the *t*-statistic for each model parameter is used as the importance measure. We used the caret package [67] in R [49] to compute these for all the classifiers. For each classification task in each scenario, we then ranked the features by importance. We show the rank data in Fig 6(b). It is apparent that weighted closeness centrality is the feature with the highest importance (lowest rank) across all scenarios, on average, with the much costlier to compute distance Laplacian-based features coming close behind.

## 4 Conclusions

We have used network science to develop tailored statistics to capture the topologies of phylogenetic trees and illustrated how they compare across different viruses and scenarios. The viruses vary in pathogenesis and their rates and modes of transmission, characteristics which ideally should be reflected in the tree shape. We find that network-science-derived statistics are complementary to asymmetry and frequency of small configurations with a tree. We have also used a tree kernel to compare trees; while it distinguishes well between three different viruses, the statistics do a better job of distinguishing the three influenza scenarios. However, no individual statistic is sufficient to consistently capture the diversity of tree topologies. For this reason, we emphasize that although the statistics we introduced may lead to biological insights about tree shapes, our main motivation was not to generate such insights, but rather, to expand the collection of statistics allowing researchers to quantify tree shapes in an efficient manner.

It is highly likely that investigators will need to use multiple summary measures to resolve biologically meaningful differences among phylogenies. This is a problem of *feature selection*. Indeed, some of the literature on phylogenetic tree topologies has focused on evaluating which summary measures are the best suited for detecting informative differences in tree topologies [5, 6, 10]. This effort makes the implicit assumption that there exists a specific subset of features that will always be a good choice for all data sets (or data for a given domain), which is not necessarily the case. There may exist summary measures that have not been formulated but would confer an optimal fit to a given dataset. Matsen [29] proposed an innovative approach to this problem, where a generalized and flexible class of tree topology statistics (which can express many established measures of tree shape) can be adapted to the data using a genetic algorithm.

A primary motivation to applying summary statistics to the shapes of phylogenetic trees is to reconstruct past evolutionary dynamics. For instance, the characteristic asymmetry of phylogenies relating infections of influenza A virus is connected to extreme variation in the persistence of viral lineages from one season to the next. This linkage between tree shape and biological processes is the foundation of phylodynamics as a rapidly emerging area of research at the interface of statistics and biology [68]. An increasing repertoire of tree shape statistics is playing a significant role in recent advances in phylodynamics. For example, spectral density profiles were recently used to assess the variation in viral fitness from time-scaled phylogenies of SARS-CoV-2 [69]. Tree shape statistics have also been employed to quantify the co-diversification of host and pathogen species [70].

The underlying evolutionary or epidemiological process may affect tree topologies, but they are likely also to be affected by sampling density and uniformity [71], geographical constraints and population structure, ecological dynamics and host contact structure (in the case of pathogens). For example, the greater asymmetry in the global IAV phylogeny relative to the other trees (as revealed by imbalance statistics such as Colless’ index) may be caused by narrowing the temporal (*e.g.*, five-year tree) or spatial (*e.g.*, USA tree) scales of sampling. Our results point to the important role of tree topologies as a vehicle for obtaining information about some of these underlying processes. Topological summary features are readily interpretable, easy to compute and scale readily to large trees, even when they do not necessarily provide direct insights into the underlying evolutionary or diversification processes. The network-based and spectral statistics we have described also generalize naturally to phylogenetic networks.

On the other hand, each feature can capture only a narrowly-defined aspect of tree shape, such as imbalance. This could potentially be overcome by using multiple features, although correlations among features may lead to diminishing returns with the addition of new features. Features can also be difficult to compare among trees of different sizes (numbers of tips), and despite attempts to derive normalized statistics they remain sensitive to size. However, Pompei and colleagues [13] have proposed a randomization procedure to derive the null distribution of summary features as a possible resolution of this problem.

## Supporting information

S1 File Supporting information for network science inspires novel tree shape statistics. (PDF)

## References

[1] Stam E. Does imbalance in phylogenies reflect only bias? Evolution. 2002;56(6):1292–1295. doi: 10.1111/j.0014-3820.2002.tb01440.x 12144028

[2] Slowinski J. Probabilities of n-Trees Under Two Models: A Demonstration that Asymmetrical Interior Nodes are not Improbable. Syst Zool. 1990;39(1):89–94. doi: 10.2307/2992212

[3] Guyer C, Slowinski J. Adaptive Radiation and the Topology of Large Phylogenies. Evolution. 1993;47(1):253–263. doi: 10.1111/j.1558-5646.1993.tb01214.x 28568105

[4] Purvis A, Fritz SA, Rodríguez J, Harvey PH, Grenyer R. The shape of mammalian phylogeny: patterns, processes and scales. Philosophical Transactions of the Royal Society B: Biological Sciences. 2011;366(1577):2462–2477. doi: 10.1098/rstb.2011.0025 21807729 PMC3138615

[5] Kirkpatrick M, Slatkin M. Searching for evolutionary patterns in the shape of a phylogenetic tree. Evolution. 1993;p. 1171–1181. doi: 10.1111/j.1558-5646.1993.tb02144.x 28564277

[6] Mooers A, Heard S. Inferring evolutionary process from phylogenetic tree shape. Quarterly Review of Biology. 1997;. doi: 10.1086/419657

[7] Blum MG, François O. Which random processes describe the tree of life? A large-scale study of phylogenetic tree imbalance. Systematic Biology. 2006;55(4):685–691. doi: 10.1080/10635150600889625 16969944

[8] Wu T, Choi K. On joint subtree distributions under two evolutionary models. Theor Popul Biol. 2015;108:13–23. 26607430 10.1016/j.tpb.2015.11.004

[9] Aldous D. Probability Distributions on Cladograms. In: Random Discrete Structures. The IMA Volumes in Mathematics and its Applications. Springer New York; 1996. p. 1–18.

[10] Agapow PM, Purvis A. Power of eight tree shape statistics to detect nonrandom diversification: a comparison by simulation of two models of cladogenesis. Syst Biol. 2002;51(6):866–72. doi: 10.1080/10635150290102564 12554452

[11] Fusco G, Cronk Q. A new method for evaluating the shape of large phylogenies. J Theor Biol. 1995;175(2):235–243. doi: 10.1006/jtbi.1995.0136

[12] Aldous DJ. Stochastic Models and Descriptive Statistics for Phylogenetic Trees, from Yule to Today. Stat Sci. 2001;16(1):23–34. doi: 10.1214/ss/998929474

[13] Pompei S, Loreto V, Tria F. Phylogenetic properties of RNA viruses. PLOS One. 2012;7(9):e44849. doi: 10.1371/journal.pone.0044849 23028645 PMC3447819

[14] Stich M, Manrubia S. Topological properties of phylogenetic trees in evolutionary models. Eur Phys J B. 2009;70(4):583–592. doi: 10.1140/epjb/e2009-00254-8

[15] Manceau M, Lambert A, Morlon H. Phylogenies support out-of-equilibrium models of biodiversity. Ecol Lett. 2015;18(4):347–356. doi: 10.1111/ele.12415 25711418

[16] Goloboff PA, Arias JS, Szumik CA. Comparing tree shapes: beyond symmetry. Zoologica Scripta. 2017;46(5):637–648. doi: 10.1111/zsc.12231

[17] Poon AF. Phylodynamic Inference with Kernel ABC and Its Application to HIV Epidemiology. Mol Biol Evol. 2015;32(9):2483–2495. doi: 10.1093/molbev/msv123 26006189 PMC4540972

[18] Saulnier E, Gascuel O, Alizon S. Inferring epidemiological parameters from phylogenies using regression-ABC: A comparative study. PLoS Comput Biol. 2017;13(3):e1005416. doi: 10.1371/journal.pcbi.1005416 28263987 PMC5358897

[19] Giardina F, Romero-Severson EO, Albert J, Britton T, Leitner T. Inference of Transmission Network Structure from HIV Phylogenetic Trees. PLOS Computational Biology. 2017;13(1):1–22. doi: 10.1371/journal.pcbi.1005316 28085876 PMC5279806

[20] Rosenberg NA. The mean and variance of the numbers of r-pronged nodes and r-caterpillars in Yule-generated genealogical trees. Annals of Combinatorics. 2006;10(1):129–146. doi: 10.1007/s00026-006-0278-6

[21] Colless DH. Relative symmetry of cladograms and phenograms: an experimental study. Syst Biol. 1995;. doi: 10.2307/2413487

[22] Sackin M. “Good” and “bad” phenograms. Systematic Biology. 1972;21(2):225–226. doi: 10.1093/sysbio/21.2.225

[23] Matsen F. A geometric approach to tree shape statistics. Syst Biol. 2006;55(4):652–661. doi: 10.1080/10635150600889617 16969941

[24] Huber KT, Spillner A, Suchecki R, Moulton V. Metrics on Multilabeled Trees: Interrelationships and Diameter Bounds. IEEE/ACM Trans Comput Biol Bioinformatics. 2011;8(4):1029–1040. doi: 10.1109/TCBB.2010.122 21116046

[25] Poon AF, Walker LW, Murray H, McCloskey RM, Harrigan PR, Liang RH. Mapping the shapes of phylogenetic trees from human and zoonotic RNA viruses. PLoS One. 2013;8(11):e78122. doi: 10.1371/journal.pone.0078122 24223766 PMC3815201

[26] Lewitus E, Morlon H. Characterizing and comparing phylogenies from their Laplacian spectrum. Systematic Biology. 2016;65(3):495–507. doi: 10.1093/sysbio/syv116 26658901

[27] Colijn C, Plazzotta G. A Metric on Phylogenetic Tree Shapes. Systematic Biology. 2018;67:113–126. doi: 10.1093/sysbio/syx046 28472435 PMC5790134

[28] Csilléry K, Blum MGB, Gaggiotti OE, François O. Approximate Bayesian Computation (ABC) in practice. Trends in Ecology & Evolution. 2010;25(7):410–418. doi: 10.1016/j.tree.2010.04.001 20488578

[29] Matsen F. Optimization over a class of tree shape statistics. IEEE/ACM Transactions on Computational Biology and Bioinformatics (TCBB). 2007;4(3):506–512. doi: 10.1109/tcbb.2007.1020 17666770

[30] Lewis TG. Network Science: Theory and Applications. Wiley; 2009.

[31] Newman M, Barabási AL, Watts DJ. The Structure and Dynamics of Networks. The Princeton Press; 2006.

[32] Hendy MD, Penny D. Spectral analysis of phylogenetic data. Journal of Classification. 1993;10(1):5–24. doi: 10.1007/BF02638451

[33] McKay BD. On the spectral characterisation of trees. Ars Combinatoria. 1977;3:219–232.

[34] Matsen FA, Evans SN. Ubiquity of synonymity: almost all large binary trees are not uniquely identified by their spectra or their immanantal polynomials. Algorithms for Molecular Biology. 2012;14(7). doi: 10.1186/1748-7188-7-14 22613173 PMC3393622

[35] Bollobás B. Modern Graph Theory. Springer; 2013.

[36] Godsil C, Royle G. Algebraic Graph Theory. Springer; 2001.

[37] Chung FRK. Spectral Graph Theory. University of Pennsylvania—AMS; 1997.

[38] Foley B, Leitner T, Apetrei C, Hahn B, Mizrachi I, Mullins J, et al. HIV Sequence Compendium 2013. Los Alamos National Laboratory, NM; 2013. LA-UR 13-26007.

[39] Kosakovsky Pond SL, Posada D, Stawiski E, Chappey C, Poon AFY, Hughes G, et al. An Evolutionary Model-Based Algorithm for Accurate Phylogenetic Breakpoint Mapping and Subtype Prediction in HIV-1. PLOS Computational Biology. 2009;5(11):1–21. doi: 10.1371/journal.pcbi.1000581 19956739 PMC2776870

[40] Benson DA, Karsch-Mizrachi I, Lipman DJ, Ostell J, Wheeler DL. GenBank. Nucleic Acids Research. 2005;33:D34–D38. doi: 10.1093/nar/gki063 15608212 PMC540017

[41] Stamatakis A. RAxML version 8: a tool for phylogenetic analysis and post-analysis of large phylogenies. Bioinformatics. 2014;30(9):1312–1313. doi: 10.1093/bioinformatics/btu033 24451623 PMC3998144

[42] Wolf E, Herbeck J, Van Rompaey S, Kitahata M, Thomas K, Pepper G, et al. Phylogenetic evidence of HIV-1 transmission between adult and adolescent men who have sex with men. AIDS Research and Human Retroviruses. 2017;33:318–22. doi: 10.1089/aid.2016.0061 27762596 PMC5372772

[43] Novitsky V, Bussmann H, Logan A, Moyo S, van Widenfelt E, Okui L, et al. Phylogenetic relatedness of circulating HIV-1C variants in Mochudi, Botswana. PLoS One. 2013;8:e80589. doi: 10.1371/journal.pone.0080589 24349005 PMC3859477

[44] Hunt G, Ledwaba J, Salimo A, Kalimashe M, Singh B, Puren A, et al. Surveillance of transmitted HIV-1 drug resistance in 5 provinces in South Africa in 2011. Communicable Diseases Surveillance Bulletin. 2013;11:122–124.

[45] Katoh K, Misawa K, Kuma K, Miyata T. MAFFT: a novel method for rapid multiple sequence alignment based on fast Fourier transform. Nucleic Acids Research. 2002;30(14):3059–3066. doi: 10.1093/nar/gkf436 12136088 PMC135756

[46] Trifinopoulos J, Nguyen LT, von Haeseler A, Minh BQ. W-IQ-TREE: a fast online phylogenetic tool for maximum likelihood analysis. Nucleic Acids Research. 2016;44:W232–W235. doi: 10.1093/nar/gkw256 27084950 PMC4987875

[47] Flouri T, Izquierdo-Carrasco F, Darriba D, Aberer AJ, Nguyen LT, Minh BQ, et al. The Phylogenetic Likelihood Library. Systematic Biology. 2015;64(2):356. doi: 10.1093/sysbio/syu084 25358969 PMC4380035

[48] Paradis E, Claude J, Strimmer K. APE: analyses of phylogenetics and evolution in R language. Bioinformatics. 2004;20:289–290. doi: 10.1093/bioinformatics/btg412 14734327

[49] R Core Team. R Core Team, editor. R: A Language and Environment for Statistical Computing. Vienna, Austria: R Foundation for Statistical Computing; 2016.

[50] Bortolussi N, Durand E, Blum MGB, François O. apTreeshape: statistical analysis of phylogenetic tree shape. Bioinformatics. 2006;22(3):363–364. doi: 10.1093/bioinformatics/bti798 16322049

[51] Stadler T. Simulating Trees with a Fixed Number of Extant Species. Systematic Biology. 2011;60(5):676–684. doi: 10.1093/sysbio/syr029 21482552

[52] McKenzie A, Steel M. Distributions of cherries for two models of trees. Mathematical Biosciences. 2000;164(1):81–92. doi: 10.1016/S0025-5564(99)00060-7 10704639

[53] Colijn C, Gardy J. Phylogenetic tree shapes resolve disease transmission patterns. Evol Med Public Health. 2014;2014(1):96–108. doi: 10.1093/emph/eou018 24916411 PMC4097963

[54] Norström MM, Prosperi MCF, Gray RR, Karlsson AC, Salemi M. PhyloTempo: A Set of R Scripts for Assessing and Visualizing Temporal Clustering in Genealogies Inferred from Serially Sampled Viral Sequences. Evol Bioinform Online. 2012;8:261–269. doi: 10.4137/EBO.S9738 22745529 PMC3382462

[55] Newman MEJ. Networks: An introduction. Oxford University Press; 2010.

[56] Newman MEJ. Analysis of weighted networks. Phys Rev E. 2004;70:056131. doi: 10.1103/PhysRevE.70.056131 15600716

[57] Mohar B, Pisanski T. How to compute the Wiener index of a graph. Journal of Mathematical Chemistry. 1988;2:267–277. doi: 10.1007/BF01167206

[58] Entringer RC, Jackson DE, Snyder DA. Distance in graphs. Czechoslovak Math J. 1976;26:283–296. doi: 10.21136/CMJ.1976.101401

[59] Wang W, Tang CY. Distributed computation of node and edge betweenness on tree graphs. In: 52nd IEEE Conference on Decision and Control; 2013. p. 43–48.

[60] Brandes U. A faster algorithm for betweenness centrality. Journal of Mathematical Sociology. 2001;25(2):163–177. doi: 10.1080/0022250X.2001.9990249

[61] Bergamini E, Borassi M, Crescenzi P, Marino A, Meyerhenke H. Computing Top-*k* Closeness Centrality Faster in Unweighted Graphs. In: 2016 Proceedings of the Eighteenth Workshop on Algorithm Engineering and Experiments (ALENEX). SIAM; 2016. p. 68–80.

[62] Wang W, Tang CY. Distributed computation of classic and exponential closeness on tree graphs. In: Proceedings of the American Control Conference. IEEE; 2014. p. 2090–2095.

[63] Morlon H, Condamine F, Lewitus E, Manceau M. RPANDA: an R package for macroevolutionary analyses on phylogenetic trees. Methods in Ecology and Evolution. 2016;R package version 1.0. doi: 10.1111/2041-210X.12526

[64] Grenfell BT, Pybus OG, Gog JR, Wood JLN, Daly JM, Mumford JA, et al. Unifying the epidemiological and evolutionary dynamics of pathogens. Science. 2004;303(5656):327–32. doi: 10.1126/science.1090727 14726583

[65] Mann HB, Whitney DR. On a Test of Whether one of Two Random Variables is Stochastically Larger than the Other. The Annals of Mathematical Statistics. 1947;18(1):50–60. doi: 10.1214/aoms/1177730491

[66] Dunn OJ. Estimation of the Medians for Dependent Variables. The Annals of Mathematical Statistics. 1959;30(1):192–197. doi: 10.1214/aoms/1177706374

[67] Kuhn M. Building Predictive Models in R Using the caret Package. Journal of Statistical Software, Articles. 2008;28(5):1–26.

[68] Volz EM, Pond SLK, Ward MJ, Brown AJL, Frost SD. Phylodynamics of infectious disease epidemics. Genetics. 2009;183(4):1421–1430. doi: 10.1534/genetics.109.106021 19797047 PMC2787429

[69] Dearlove B, Lewitus E, Bai H, Li Y, Reeves DB, Joyce MG, et al. A SARS-CoV-2 vaccine candidate would likely match all currently circulating variants. Proceedings of the National Academy of Sciences. 2020;117(38):23652–23662. doi: 10.1073/pnas.2008281117 32868447 PMC7519301

[70] Avino M, Ng GT, He Y, Renaud MS, Jones BR, Poon AF. Tree shape-based approaches for the comparative study of cophylogeny. Ecology and evolution. 2019;9(12):6756–6771. doi: 10.1002/ece3.5185 31312429 PMC6618157

[71] Frost SD, Volz EM. Modelling tree shape and structure in viral phylodynamics. Philosophical Transactions of the Royal Society B: Biological Sciences. 2013;368(1614):20120208. doi: 10.1098/rstb.2012.0208 23382430 PMC3678332

