# Supplementary material for: Network science inspires novel tree shape statistics
Source: PLoS One. 2021 Dec 23;16(12):e0259877. doi: 10.1371/journal.pone.0259877 (PMC8699983; doi:10.1371/journal.pone.0259877)
Supplement: S1 File — (PDF) [file pone.0259877.s001.pdf]

Supporting information for Network science inspires novel tree shape statistics

1 GenBank accession numbers for outgroups

Virus	Outgroup	GenBank accession number
HIV-1 subtype B	a subtype D sequence	AY071949
Dengue virus serotype 4	isolate from the Philippines (1956)	U18433
Measles virus	a genotype D6 sequence	AY523581

Table S1: Accession numbers for outgroups

2 p-values for the statistics used to compare viruses or scenarios

The p-values come from the Mann-Whitney U test for each statistic in a pairwise comparison; the values passing Bonferroni correction for multiple testing at the $\alpha = 0.05$ level are in bold.

	fiveyrflu-globflu	globflu-usaflu	fiveyrflu-usaflu	Dengue-HIV	Dengue-Measles	HIV-Measles	Yule-Biased	Yule-R0_1.5	Yule-R0_3	Biased-R0_1.5	Biased-R0_3	R0_1.5-R0_3
between	**1.87e-38**	**9.49e-23**	**1.73e-05**	0.116	**3.74e-05**	0.137	0.724	0.0114	0.338	0.00809	0.427	0.097
betweenW	**1.87e-38**	**9.49e-23**	**1.73e-05**	0.116	**3.74e-05**	0.137	0.724	0.0114	0.338	0.00809	0.427	0.097
closeness	**1.03e-22**	0.0357	**3.29e-25**	**1.49e-62**	**8.12e-96**	0.00267	**1.01e-30**	**1.27e-19**	**1.74e-07**	**1.09e-41**	**1.91e-40**	**5.23e-08**
closenessW	**5.71e-17**	0.546	**4.86e-14**	**1.4e-136**	**5.85e-118**	**1.91e-142**	0.001	**8.22e-109**	**5.07e-100**	**5.04e-107**	**1.83e-95**	**1.26e-07**
eigen	0.399	0.012	0.0526	0.934	0.00477	0.00112	0.0658	**2.72e-05**	0.105	0.000363	0.795	0.00789
eigenW	0.00523	0.163	0.0573	0.00169	0.432	**1.61e-18**	0.101	**1.51e-08**	**1.42e-07**	**1.52e-08**	**1.43e-07**	0.353
diameter	**7.79e-21**	0.354	**5.08e-22**	**1.46e-67**	**1.12e-74**	0.0595	**1.13e-23**	**3.42e-17**	**1.63e-06**	**1.89e-39**	**2.48e-35**	**8.73e-07**
meanpath	**2.35e-15**	0.00199	**9.51e-21**	**1.54e-81**	**2.34e-73**	0.000369	**2.03e-36**	**1.35e-21**	**1.81e-09**	**1.14e-40**	**6.41e-42**	**4.58e-09**
minAdj	0.0525	0.608	0.18	**6.87e-12**	**4.44e-06**	0.0222	**5.82e-05**	**5.36e-06**	0.0873	**1.39e-16**	**7.61e-09**	0.00262
maxAdj	**3.07e-05**	0.62	**4.22e-06**	**6.74e-64**	**2.11e-37**	**2.78e-28**	**2.9e-20**	**5.01e-13**	**4.66e-05**	**1.03e-33**	**3.95e-30**	**8.35e-05**
minLap	**6.37e-23**	**6.63e-06**	**2.41e-30**	**2.19e-39**	**3.17e-97**	0.587	**4.04e-12**	**8.29e-13**	0.00143	**7.43e-33**	**2.51e-21**	**1.21e-05**
maxLap	**9.17e-05**	0.855	**3.64e-05**	**1.47e-59**	**3.57e-27**	**1.43e-33**	**1.18e-14**	**3.6e-09**	0.00221	**3.43e-28**	**1.55e-23**	0.000958
cherries	0.515	0.00773	0.0268	**5.79e-28**	**2.35e-05**	**4.45e-20**	**1.15e-19**	**1.25e-31**	**9.33e-11**	**1.1e-55**	**3.02e-36**	**1.23e-10**
pitchforks	0.0122	0.765	0.0283	**7.42e-14**	**1.08e-13**	0.565	0.0491	0.00465	0.0103	**3.37e-06**	**1.03e-05**	0.77
doubcherries	0.458	0.0415	0.192	**1.31e-07**	0.0368	0.000347	0.000208	**2.51e-06**	0.119	**1.33e-13**	**1e-06**	0.00227
fourprong	**0.000101**	0.582	0.00295	0.774	**3.61e-08**	**1.17e-08**	0.0113	0.00175	0.284	**1.21e-07**	0.000595	0.0448
num5	0.0057	0.00411	0.962	3e-04	**2.35e-13**	**6.51e-05**	0.693	0.485	0.387	0.237	0.186	0.821
num6	0.000614	0.000228	0.505	0.000557	**2.06e-13**	**6.3e-05**	0.459	0.239	0.0362	0.662	0.177	0.363
colless	**2.36e-21**	**5.73e-20**	0.125	0.0918	**5.23e-87**	**1.52e-59**	**1.15e-28**	**1.26e-22**	**3.75e-11**	**3.46e-36**	**9.93e-38**	**3.44e-10**
sackin	**3.37e-20**	**2.29e-20**	0.0378	0.469	**4.17e-81**	**2.09e-57**	**6.98e-32**	**3.85e-22**	**7.95e-11**	**3.24e-35**	**3.82e-36**	**2.82e-10**
maxwidth	**1.19e-13**	0.238	**2.53e-17**	**2.88e-05**	**4.95e-64**	**1.65e-34**	**1.06e-29**	**5.96e-15**	**7.32e-05**	**1.01e-58**	**2.39e-42**	**4.04e-05**
maxheight	**1.86e-25**	**1.2e-12**	0.000156	**3.36e-09**	**1.11e-73**	**3.05e-47**	**3.21e-27**	**1.77e-18**	**1.04e-06**	**2.65e-38**	**2.62e-37**	**1.37e-08**
stairs	0.184	0.00291	0.0675	**1.1e-27**	**2.01e-07**	**1.39e-15**	**2.57e-14**	**1.47e-26**	**2.35e-07**	**1.22e-45**	**5.26e-26**	**1.37e-08**
delW	0.000534	1	0.000174	0.124	**5.27e-21**	**2.73e-18**	**9.47e-25**	**5.35e-10**	0.00257	**8.65e-49**	**4e-35**	0.00118
lambdaMax	0.00265	0.0566	**2.79e-05**	**4.35e-48**	**2.58e-16**	**4.44e-69**	**3.85e-07**	**1.39e-42**	**1.55e-68**	**4.74e-36**	**1.97e-56**	**9.91e-23**
asymmetry	0.000291	0.865	0.000994	**5.78e-07**	**1.25e-36**	**1.03e-46**	0.175	**1.96e-11**	**3.15e-36**	**2.38e-10**	**1.45e-34**	**1.03e-24**
kurtosis	0.014	0.891	0.00115	**2.15e-53**	**1.33e-11**	**2.08e-74**	0.91	**2.54e-53**	**3.66e-48**	**2.09e-50**	**9.32e-47**	**1.05e-12**
densityMax	0.152	0.237	0.0281	**1.1e-132**	**3.4e-99**	**2.19e-148**	0.0113	**1.11e-60**	**1.22e-54**	**1.04e-60**	**1.11e-54**	**1.16e-10**

Table S2: p-values for pairs of scenarios in Figure 2.

3 Kernel-based tree comparisons

Rather than using summary statistics to quantify differences in tree topologies, we can calculate a similarity or distance measure between trees. For example, the Robinson-Foulds distance

	Wolf-Hunt	Hunt-Novitsky	Wolf-Novitsky	Yule_300-Biased_300	Yule_300-R0_1.5_300	Yule_300-R0_3_300	Biased_300-R0_1.5_300	Biased_300-R0_3_300	R0_1.5_300-R0_3_300
between	0.613	0.0864	0.203	0.955	0.931	0.454	0.955	0.355	0.491
betweenW	0.613	0.0864	0.203	0.955	0.931	0.454	0.955	0.355	0.491
closeness	**2.68e-29**	**3.38e-05**	**3.24e-40**	**5.58e-51**	**2.93e-59**	**9.48e-38**	**1.15e-70**	**1.63e-66**	**6.47e-23**
closenessW	**1.48e-37**	**4.25e-90**	**3.93e-122**	**1.6e-05**	**1.88e-110**	**2.52e-104**	**1.07e-142**	**2.02e-151**	**1.04e-07**
eigen	0.305	0.774	0.599	0.0138	**1.75e-11**	0.000752	**5.84e-10**	0.026	0.000536
eigenW	0.479	0.0736	0.126	0.612	**7.6e-08**	**5.14e-09**	**7.6e-08**	**5.14e-09**	0.947
diameter	**5.07e-14**	0.919	**8.16e-15**	**1.01e-36**	**4.25e-42**	**3.07e-22**	**2.34e-56**	**6.84e-53**	**3.1e-18**
meanpath	**5.58e-34**	0.000281	**2.42e-45**	**7.26e-56**	**2.38e-50**	**8.81e-41**	**1.28e-59**	**8.72e-61**	**1.04e-25**
minAdj	0.000235	0.369	0.00246	**3.88e-05**	**3.49e-28**	**4.36e-14**	**7.71e-42**	**6.38e-27**	**4.32e-06**
maxAdj	**1.92e-37**	**3.46e-11**	**4.62e-60**	**8.73e-39**	**2.17e-60**	**1.24e-35**	**4.59e-67**	**4.83e-56**	**3.41e-21**
minLap	**5.28e-13**	0.0089	**6.73e-19**	**1.6e-26**	**1.24e-33**	**1.26e-15**	**8.98e-64**	**6.49e-55**	**2.23e-10**
maxLap	**5.29e-35**	**1.06e-10**	**2.35e-57**	**7.6e-32**	**1.13e-55**	**6.74e-30**	**2.21e-63**	**2.75e-51**	**2.67e-19**
cherries	0.0149	0.00883	**3.52e-07**	**3.5e-39**	**8.79e-70**	**4.92e-37**	**3.2e-99**	**1.23e-73**	**2.93e-20**
pitchforks	**8.5e-05**	0.00429	0.226	**0.000119**	**5.88e-15**	**5.75e-07**	**6.3e-32**	**2.22e-20**	0.000248
doubcherries	0.00515	0.0178	**3.87e-07**	**1.56e-08**	**3.4e-14**	0.000252	**2.95e-32**	**2.86e-19**	**1.61e-06**
fourprong	0.00603	0.00224	0.799	**1.41e-07**	0.0269	0.032	**2.67e-12**	**6.59e-12**	0.972
num5	**6.35e-05**	0.324	**2.7e-07**	0.517	0.02	0.641	0.12	0.842	0.0669
num6	0.317	0.00385	**1.35e-05**	0.118	0.416	0.902	0.468	0.0725	0.325
colless	**1.37e-05**	**1.12e-14**	**9.28e-24**	**4.94e-31**	**3.56e-36**	**6.08e-26**	**4.88e-44**	**2.58e-45**	**2.95e-18**
sackin	**4.45e-06**	**8.42e-14**	**2.15e-23**	**4.42e-35**	**4.05e-35**	**3.86e-27**	**4.32e-43**	**9.3e-46**	**6.1e-18**
maxwidth	**4.52e-06**	0.000964	0.0565	**1.35e-47**	**5.52e-31**	**6.92e-08**	**4.03e-93**	**2.82e-68**	**2.42e-15**
maxheight	0.655	**2.3e-13**	**9.51e-13**	**6.52e-32**	**1.19e-37**	**1.25e-16**	**4.68e-51**	**1.54e-45**	**2.8e-18**
stairs	0.000931	0.00214	**7.94e-10**	**8.73e-28**	**5.75e-58**	**1.97e-25**	**4.01e-87**	**5.18e-61**	**2.4e-20**
delW	0.00287	0.119	0.105	**6.46e-47**	**5.19e-22**	**3.22e-07**	**1.86e-81**	**2.14e-65**	**3.27e-08**
lambdaMax	0.122	**2.19e-37**	**2.63e-46**	**6.85e-08**	**2.14e-64**	**6.19e-79**	**1.71e-53**	**5.17e-64**	**1.07e-22**
asymmetry	**1.09e-34**	**2.98e-84**	**6.55e-105**	0.000942	**6.06e-29**	**9.41e-36**	**4.44e-25**	**3.74e-34**	**5.38e-20**
kurtosis	**1.71e-102**	**3.2e-110**	**1.35e-156**	0.0436	**1.14e-58**	**8.03e-59**	**1.58e-55**	**2.88e-57**	**8.21e-25**
densityMax	**4.56e-40**	0.00644	**7.98e-55**	0.0114	**9.21e-72**	**2.61e-72**	**9.09e-72**	**2.56e-72**	**4.45e-15**

Table S3: p-values for pairs of scenarios in Figure 3.

measure is an edit distance between trees with the same labels (*i.e.*, relating the same taxa). The resulting distance matrix for a set of tree topologies can then be interpreted as a measure space for supervised or unsupervised classifiers [1]. However, the requirement for shared labels in the Robinson-Foulds distance prevents its application to the present problem of comparing phylogenies from different viruses.

To overcome this limitation, we previously adapted a kernel method from natural language processing [2] to provide a similarity measure that operates on both the topology and branch lengths of trees [3]. Every tree is comprised of a large number of subset trees that can each act as a feature. A subset tree is a contiguous set of branches rooted at an internal node of its parent tree, which does not necessarily include all descendants of that internal node. In other words, the subset tree does not have to extend out to the tips of the parent tree.

A subset tree is completely defined by its branching order if we rotate branches of the tree (so-called "ladderization") so that all branching events occur preferentially to one side. For a tree of even modest size, the number of all possible subset trees is extremely large; the space of all possible subset trees is even more immense. Clearly, it is not feasible to exhaustively enumerate the appearance of every possible subset tree for a given observed tree.

The kernel trick is a well-established technique in machine learning that provides an efficient way to compare trees with respect to their subset trees by limiting the comparison to those features that appear in one or both trees [3]. Calculating the inner products over this restricted set of features for every pair of trees yields a distance matrix which defines a projection of the trees into a high dimensional space with convenient properties for machine learning.

We applied this kernel method to the data sets examined above with the following parameter settings: decay factor $\lambda = 0.2$; radial basis function variance $\sigma = 2$.

Figure S1 represents a set of principal component analysis (PCA) plots that illustrate the separation of HIV, Dengue, and Measles virus phylogenies into distinct clusters in the space defined by the tree kernel. The HIV trees are separated from the others along the first principal component that explained roughly 75% of the overall variation. Dengue and Measles virus trees can only be separated into distinct clusters by the third principal component.

For comparison, the first two principal components of the tree shape statistics shown in Figure S2 are already able to separate the three viruses. The dots represent individual trees; the arrows point in the direction of maximum multiple correlation with the principal components for each tree shape statistic, while their length indicates the strength of this multiple correlation. Once again, we see that the network science-based statistics are over-represented among the longest arrows.

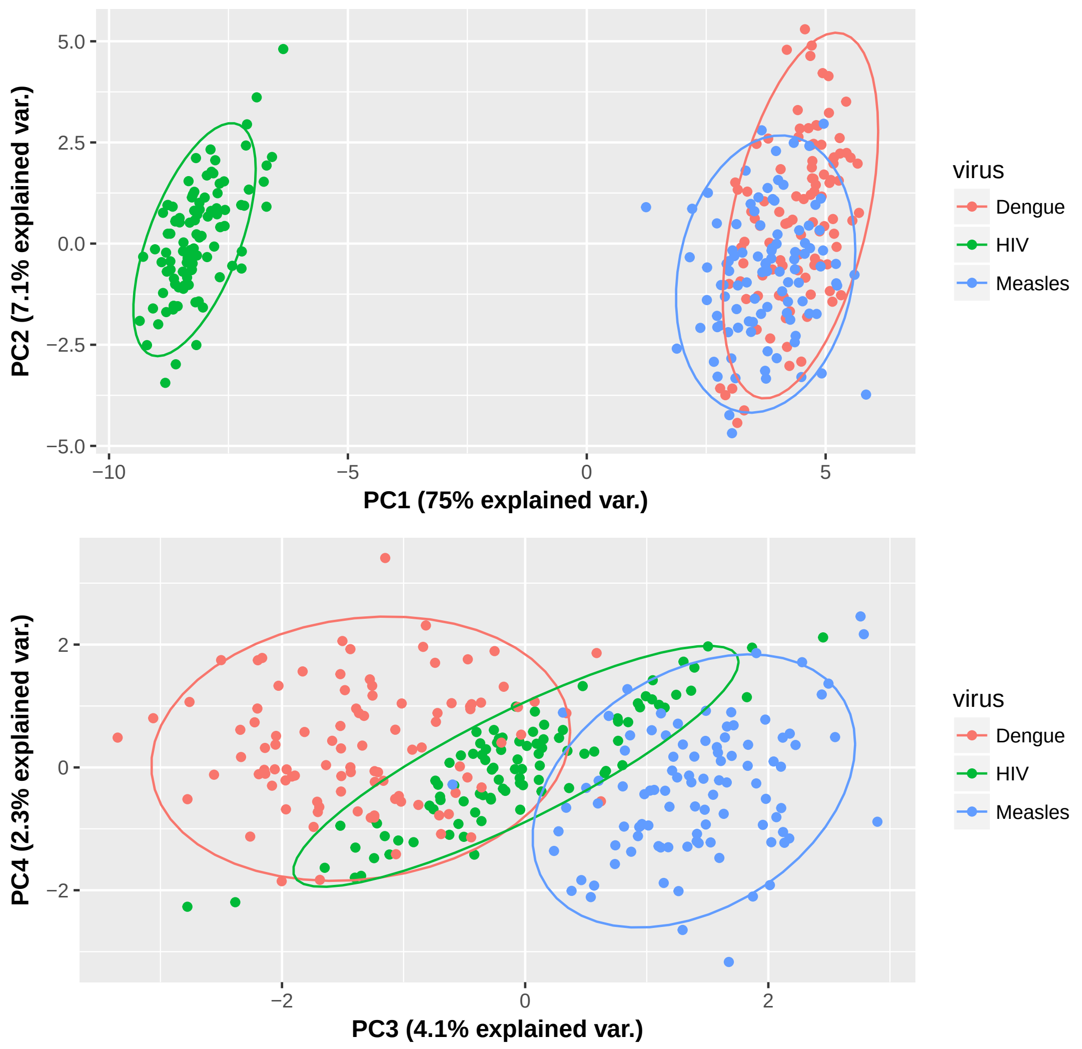

Figure S1: Kernel PCA plots of first four principal components illustrating the separation of HIV (green), Dengue (red) and Measles (blue) virus phylogenies by a tree kernel method.

Furthermore, applying the same method to trees from the three different samples of influenza A virus outbreaks was less promising (Figure S4).

While it is possible that adjusting the kernel tuning parameters (λ and σ) could yield better results, we would risk over-fitting this method to these data. In contrast, Colless and Sackin imbalance separate global flu from the other two groups, while closeness, diameter and mean path separate the five-year flu trees from the global and USA groups. A combination of these summary statistics separate the three groups, as shown in Figure S3 below.

We observed a similar situation in the case of the other three datasets, in which the first three principal components of the kernel-based method are sometimes needed to separating the different categories or scenarios (Figures S5, S7 and S8, while only the first two principal components of the tree shape statistics considered in this paper are usually sufficient (Figures S6 and S9). However, both methods are clearly able to identify enough signal in the data to separate the categories or scenarios.

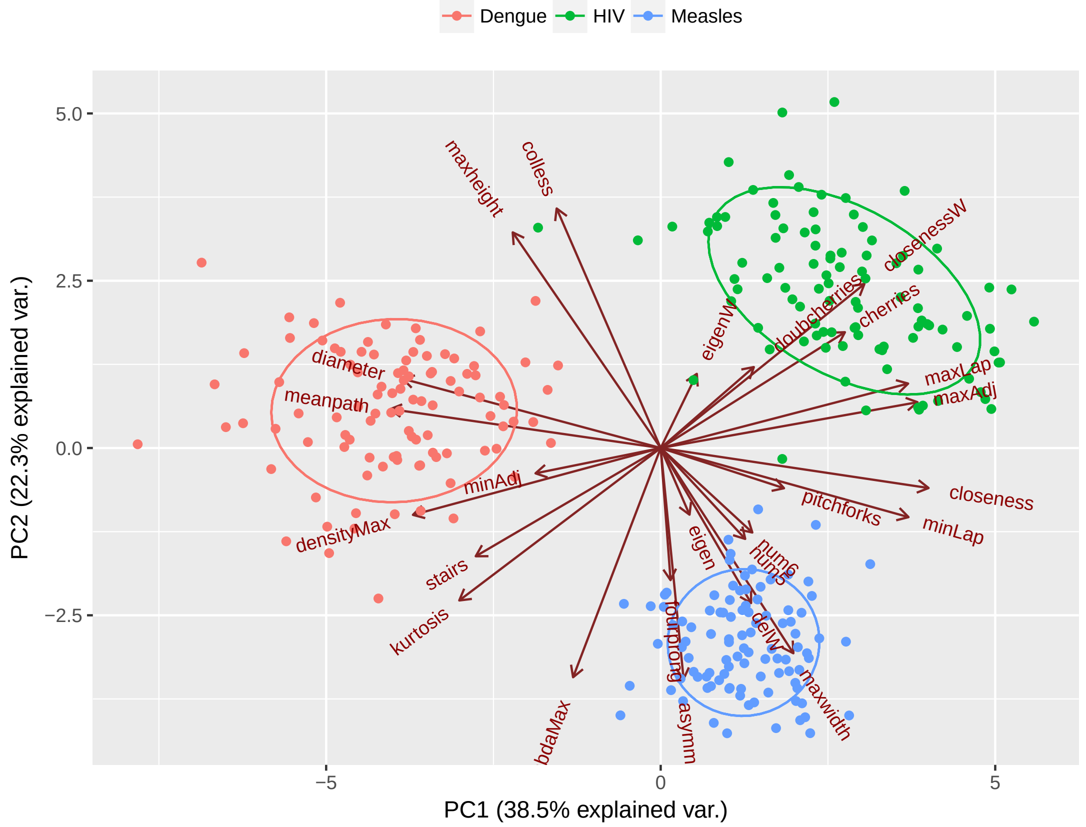

Figure S2: PCA biplots of first two principal components illustrating the separation of HIV (green), Dengue (red) and Measles (blue) virus phylogenies by the shape statistics.

4 Calculating the (degree) assortativity of a phylogenetic tree

Consider a phylogenetic tree on n tips as a graph. Its degree assortativity is the Pearson correlation between the degrees of the sources (heads) and targets (tails) of its edges. We direct each edge away from the root, towards the tips. We show below that for $n \geq 4$, only two values of the assortativity coefficient can occur (only one such value can obviously occur for $n \leq 3$).

We make use of the following formula for the Pearson correlation between $\vec{x} \in \mathbb{R}^N$ and $\vec{y} \in \mathbb{R}^N$:

$$r_{xy} := \frac{N\sum_{i=1}^{N} x_i y_i - \sum_{i=1}^{N} x_i \sum_{i=1}^{N} y_i}{\sqrt{N\sum_{i=1}^{N} x_i^2 - (\sum_{i=1}^{N} x_i)^2}\sqrt{N\sum_{i=1}^{N} y_i^2 - (\sum_{i=1}^{N} y_i)^2}}.$$

Based on this formula, we note that only two options can occur; the first option is when the root's children are both internal nodes, and the second option is when one of its children is a tip and the other, an internal node (which occurs when, for instance, there is an outgroup in the data). In the first case, we use

$$\vec{x} = (\underbrace{3, \ldots, 3}_{n \text{ times}}; 2, 2; \underbrace{3, \ldots, 3}_{n-4 \text{ times}})$$
$$\vec{y} = (\underbrace{1, \ldots, 1}_{n \text{ times}}; 3, 3; \underbrace{3, \ldots, 3}_{n-4 \text{ times}})$$

since there are exactly n edges going from an internal node to a tip, 2 edges from the root to an internal node, and $n - 4$ edges from an internal node to another internal node. An easy

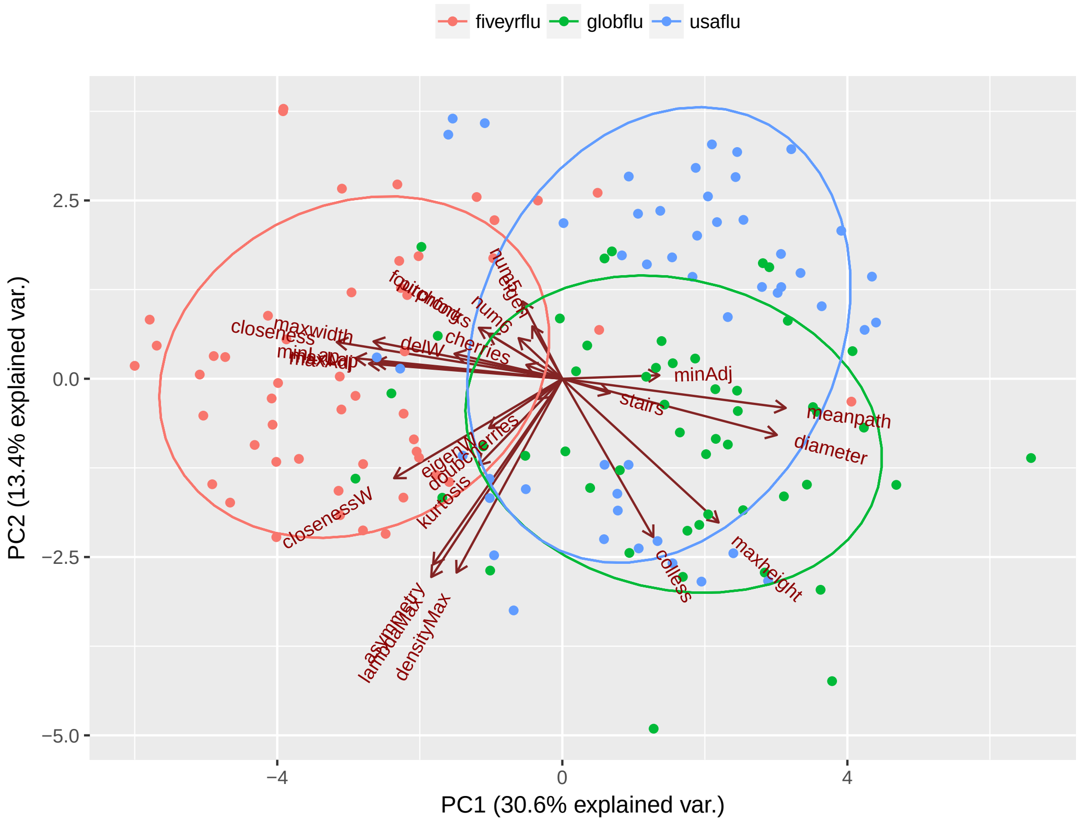

Figure S3: PCA biplots illustrating the separation among influenza A virus phylogenies sampled at global (green), five-year (red) and regional (United States, blue) scopes by using tree shape statistics

calculation then yields

$$A_1(n) := \frac{-\sqrt{n}}{n-2} \text{ and } A_1'(n) := \frac{-(n^2+2n+1)}{3n^2-4n-3},$$

where A_1 denotes the *directed* degree assortativity, calculated by substituting $\vec{x}$ and $\vec{y}$ directly into the formula above, while A_1' denotes the *undirected* degree assortativity, calculated by substituting $[\vec{x}\vec{y}]$ and $[\vec{y}\vec{x}]$ into it, where $[\vec{u}\vec{v}]$ denotes the concatenation of two vectors. The latter occurs because in the undirected case, each directed edge (ij) is counted twice, once as an edge from i to j and once as an edge from j to i.

In the second case, where the root has a tip as one of its children, the degree sequences are

$$\vec{x} = (\underbrace{3,\ldots,3}_{n-1 \text{ times}}; 2, 2; \underbrace{3,\ldots,3}_{n-3 \text{ times}})$$

$$\vec{y} = (\underbrace{1,\ldots,1}_{n-1 \text{ times}}; 1, 3; \underbrace{3,\ldots,3}_{n-3 \text{ times}})$$

and the corresponding values are

$$A_2(n) := \frac{-1}{\sqrt{n}(n-2)} \text{ and } A_2'(n) := \frac{-(n^2-2n+5)}{3n^2-4n-3}.$$

We note that $\lim_{n\to\infty} A_1(n) = 0 = \lim_{n\to\infty} A_2(n)$ and $\lim_{n\to\infty} A_1'(n) = -1/3 = \lim_{n\to\infty} A_2'(n)$.

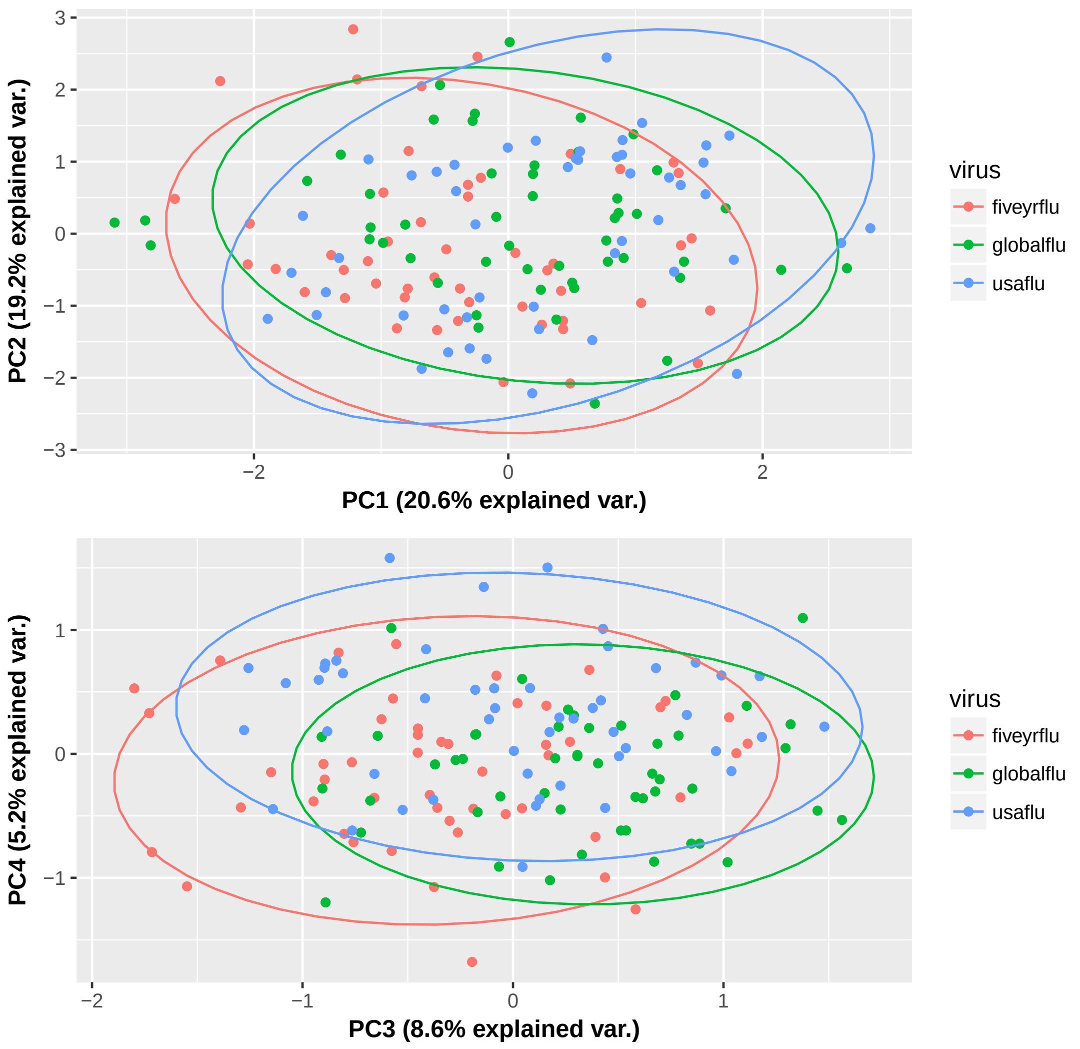

Figure S4: Kernel PCA plots illustrating the overall lack of separation among influenza A virus phylogenies sampled at global (green), five-year (red) and regional (United States, blue) scopes by using the tree kernel method.

5 The distribution of the diameter and the Wiener index of trees

The diameter for a phylogenetic tree on n tips nodes ranges from $\approx 2\log_2(n)$ for the maximally balanced tree to n for the double caterpillar, while the average is proportional to $\sqrt{n}$ [4]. For a specific example, the distribution of diameters for $n = 23$ tips ranges from 8 to 23, with a mean of 14.147. Figure S10 shows the distribution of all the diameters, together with one of the trees achieving the minimum and maximum values of the diameter, respectively.

The Wiener index for a phylogenetic tree on n tips ranges from $\approx 4n^2\log_2(n)$ to $\approx \frac{2}{3}n^3$, in each case to leading order, while the average is proportional to $n^2\sqrt{n}$ [5]. For a specific example, the distribution of Wiener indices for $n = 23$ tips ranges from 5382 to 8778, with a mean of 6516.541. Figure S11 shows the distribution of all the diameters, together with the trees achieving the minimum and maximum values of the Wiener index, respectively.

Figure S12 illustrates all 6 phylogenetic trees on $n = 6$ tips with their Wiener index and diameter, arranged in increasing order of the Wiener index, with ties broken by the diameter.

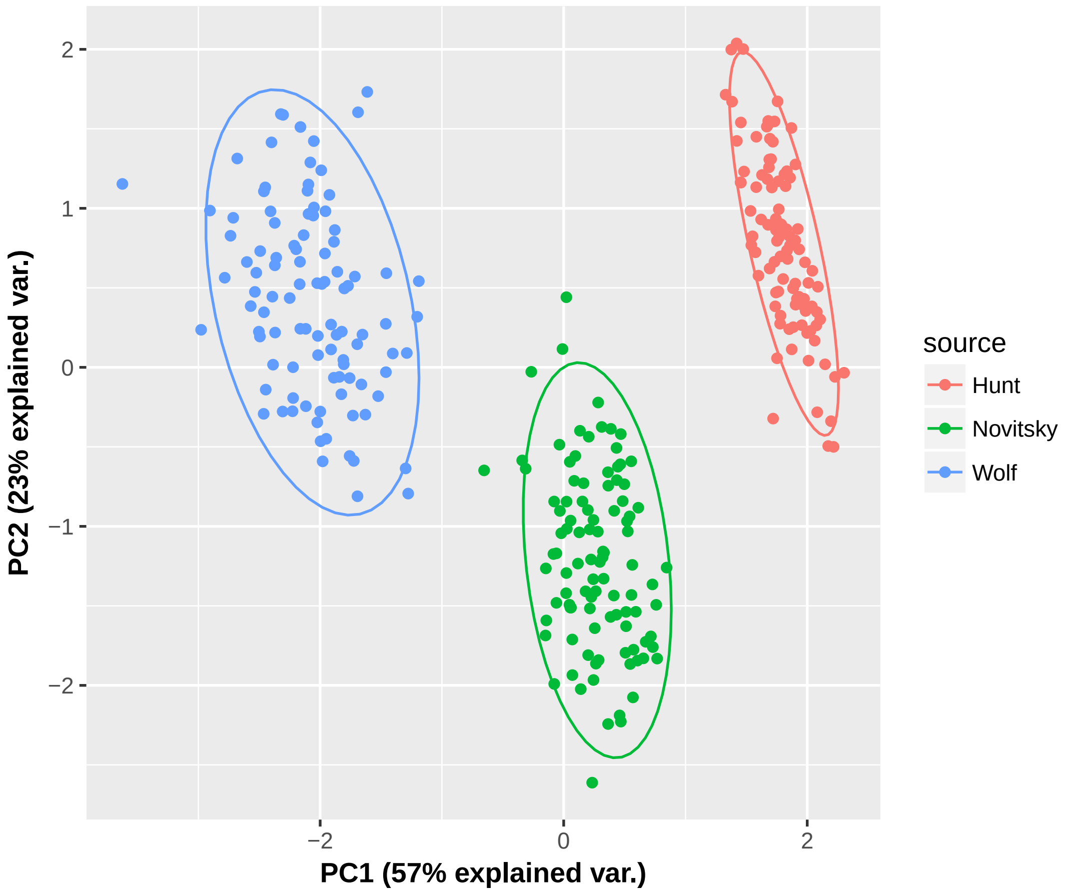

Figure S5: Kernel PCA plots illustrating the separation among HIV phylogenies sampled from a concentrated epidemic (blue), a generalized epidemic in a country (red) and in a village (green) by using the kernel method.

6 The distribution of betweenness/closeness/eigenvector centrality

The distribution of maximum betweenness centrality values for $n = 23$ tips ranges from 505 to 645, with a mean of 587.592. Figure S13 shows the distribution of all the maximum betweenness centrality values, together with one of the trees achieving the minimum and maximum values, respectively.

The distribution of maximum closeness centrality values for $n = 23$ tips ranges from 143 to 285, with a mean of 189.920. Figure S14 shows the distribution of all the maximum closeness centrality values, together with the trees achieving the minimum and maximum values, respectively.

The distribution of maximum eigenvector centrality values for $n = 23$ tips ranges from 0.2324 to 0.4336, with a mean of 0.3573. Figure S15 shows the distribution of all the maximum eigenvector centrality values, together with the trees achieving the minimum and maximum values, respectively.

Figure S16 illustrates all 6 phylogenetic trees on $n = 6$ tips with their betweenness, closeness, and eigenvector centrality, arranged in increasing order of the betweenness, with ties broken by the closeness.

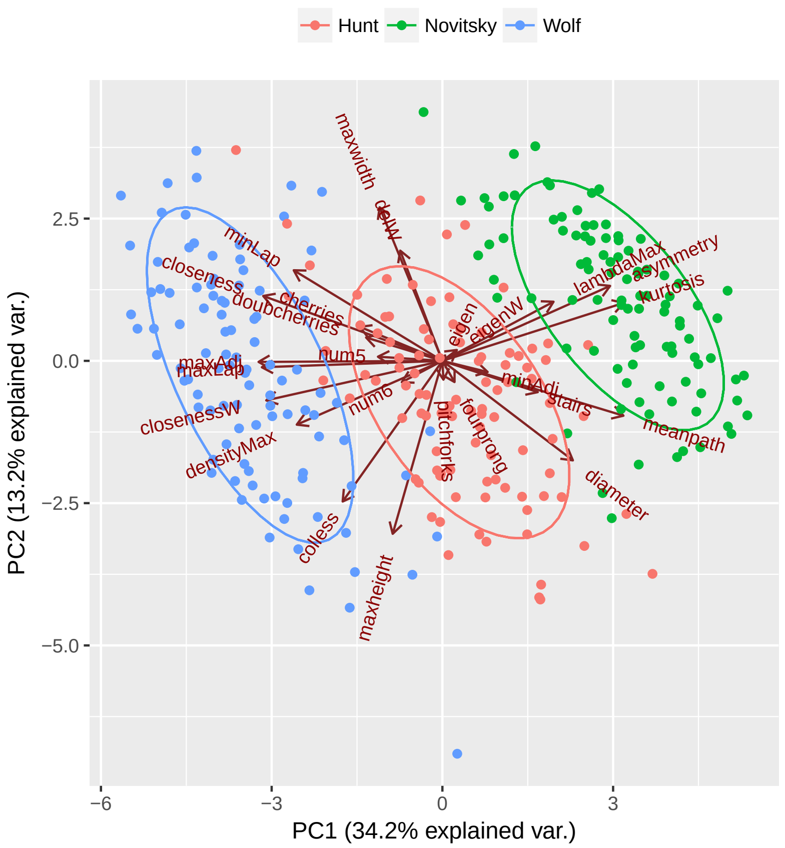

Figure S6: PCA biplots illustrating the separation among HIV phylogenies sampled from by using tree shape statistics.

7 Proof of the location of the maximum values of centralities

We now present a simple proof for the following facts, mentioned in the main text:

a. Betweenness centrality is maximized at the **tricenter** of the tree (the node which, if the tree is rerooted at it, has 3 descendant clades of sizes within one of each other), if one exists, and minimum at the tips

b. Closeness centrality is maximized at the **centroid** of the tree (the node which, if the tree is rerooted at it, has all descendant clades containing fewer than half the total nodes), and minimum at one of the tips

c. Eigenvector centrality is maximized at one of the internal nodes

Proof:

a. Let v be any node in a tree T. If n_1 and n_2 are the sizes of the left and right subtrees of v and n_0 is the number of nodes outside T_v, the clade of T subtended by v, then its betweenness centrality is $n_0 n_1 + n_0 n_2 + n_1 n_2$. We maximize this quantity subject to the constraint $n_0 + n_1 + n_2 = N - 1$ and keep in mind that all of the sizes are integers. First, if $N \equiv 1 \mod 3$, all three of these quantities can be equal to $\frac{N-1}{3}$, and this matches the best we can do even if we allow fractional values. Second, if $N \equiv 2 \mod 3$, say $N = 3K + 2$, the best option is to set one of the three values to $K + 1 = \frac{N+1}{3}$ and make the remaining two equal to $K = \frac{N-2}{3}$. Finally, if $N \equiv 0 \mod 3$, say $N = 3K$, the best option is to set two of the three values to $K = \frac{N}{3}$ and make the remaining one equal to $K - 1 = \frac{N}{3} - 1$.

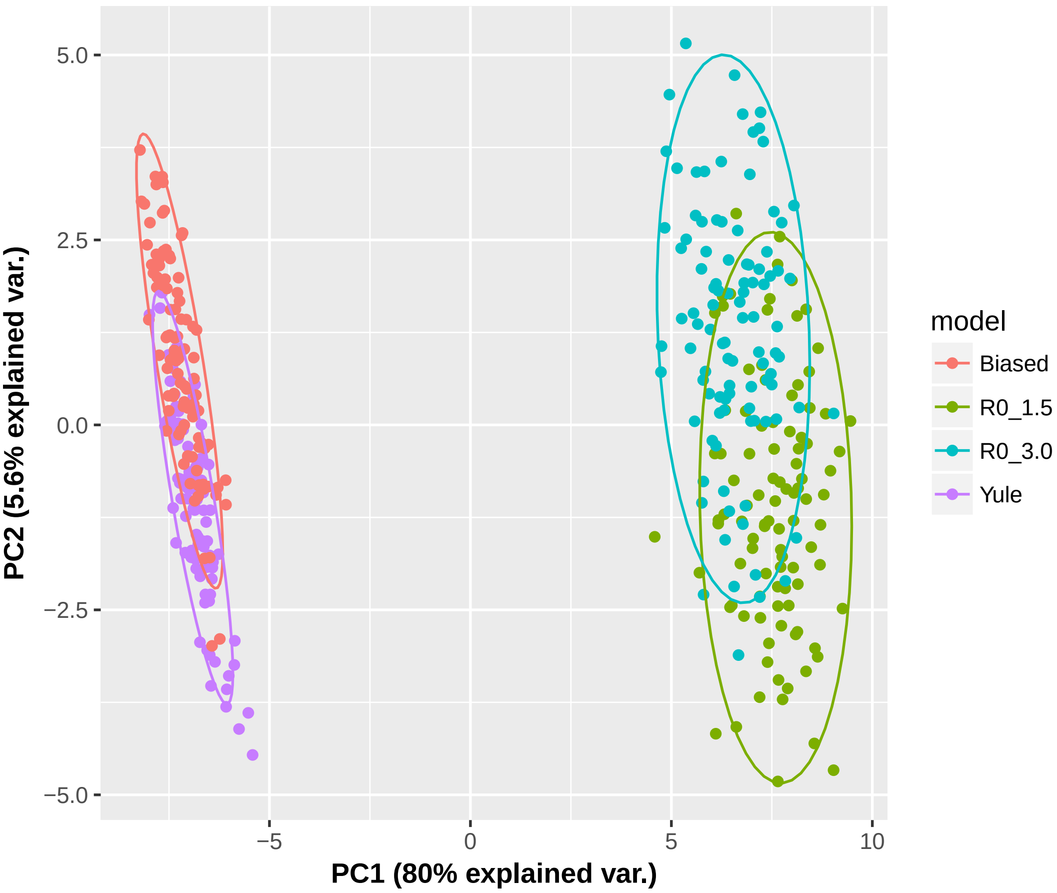

Figure S7: Kernel PCA plot illustrating the separation among phylogenies simulated from a biased model (red), a Yule model (purple) and two birth-death models (green and blue) by using the kernel method.

In summary, the maximum is attained when $|n_0 - n_1| \leq 1, |n_0 - n_2| \leq 1, |n_1 - n_2| \leq 1$, and at least one of these inequalities is strict. We call a node which satisfies this condition a **tricenter** of the tree. Not all trees have one - for instance, the complete binary tree on $n = 4$ tips does not have one. The uniqueness of a tricenter follows from the uniqueness of the centroid, proven as part of the next result. Furthermore, the value of betweenness centrality at a tricenter is close to $\frac{N^2}{3}$, while having $n_0 = 0$ (as would be the case for the root) results in a value of at most $\frac{N^2}{4}$, which is clearly suboptimal. Lastly, it is also clear that $n_1 = n_2 = 0$ (as would be the case for any tip) results in a betweenness centrality value of 0, the minimum possible.

b. Let u be the node of a phylogenetic tree T_0 that has the smallest farness; we will show that u must be a centroid, and conclude by showing that every phylogenetic tree has a unique centroid. For the first part, let us consider the tree T (which will not be binary if u is not the root) obtained by rerooting T_0 at u. Note that the distances in T are equal to the distances in T_0. To make things general we consider a tree with branch lengths (weights).

Let v be any child of u in T. Note that $d(v, x) = d(u, x) - w(uv)$ for any node x in the subtree T_v rooted at v, and $d(v, x) = d(u, x) + w(uv)$ for any node x outside T_v (including

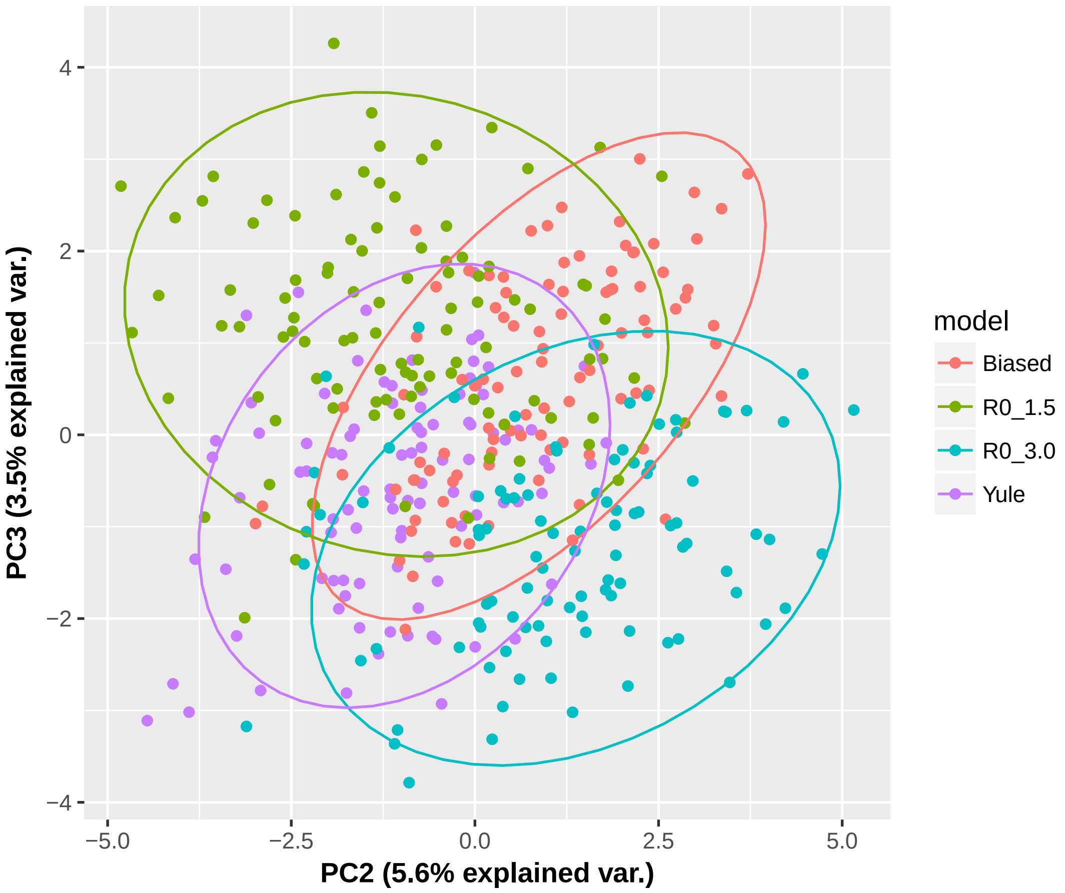

Figure S8: The same kernel PCA plot as above, components 2 and 3.

u itself). If we denote by $F(x)$ the farness of a node x, then

$$F(v) = \sum_{x \in T_v} (d(u,x) - w(uv)) + \sum_{x \notin T_v} (d(u,x) + w(uv))$$
$$= \sum_{x \in V(T)} d(u,x) + (N - 2|T_v|)w(uv) = F(u) + (N - 2|T_v|)w(uv).$$

This recurrence is in fact the basis for a linear-time algorithm for computing $F(x)$ for all nodes x in T_0, starting from the root. Recall that $w(uv) > 0$ by assumption. Therefore, in order for $F(u)$ to be the smallest farness, we must have

$$F(v) \geq F(u) \iff N - 2|T_v| \geq 0 \iff T_v \leq \frac{N-1}{2}, \tag{1}$$

since $N = 2n - 1$ is odd, and in fact, the first two inequalities must be strict. It follows that the subtree T_v of T rooted at any child v of u must contain fewer than half of all the nodes; therefore, u must satisfy the definition of a centroid. We note that two closely related results appear in the work on 1-medians of tree networks [6].

It remains to show that the centroid exists and is unique in a phylogenetic tree. For the existence, we apply a result by Camille Jordan [7] that proves the existence of a *separator node* v in any (not necessarily binary) tree T, which is a node defined by the property that any connected component obtained by removing v from T has size at most $\frac{N}{2}$, where N is the number of nodes in T. The reason that such a separator node is a centroid is that the connected components resulting from removing v are identical with the subtrees rooted at v if T is rerooted at v, and that N is odd, so $x \leq \frac{N}{2} \iff x < \frac{N}{2}$ for $x \in \mathbb{N}$.

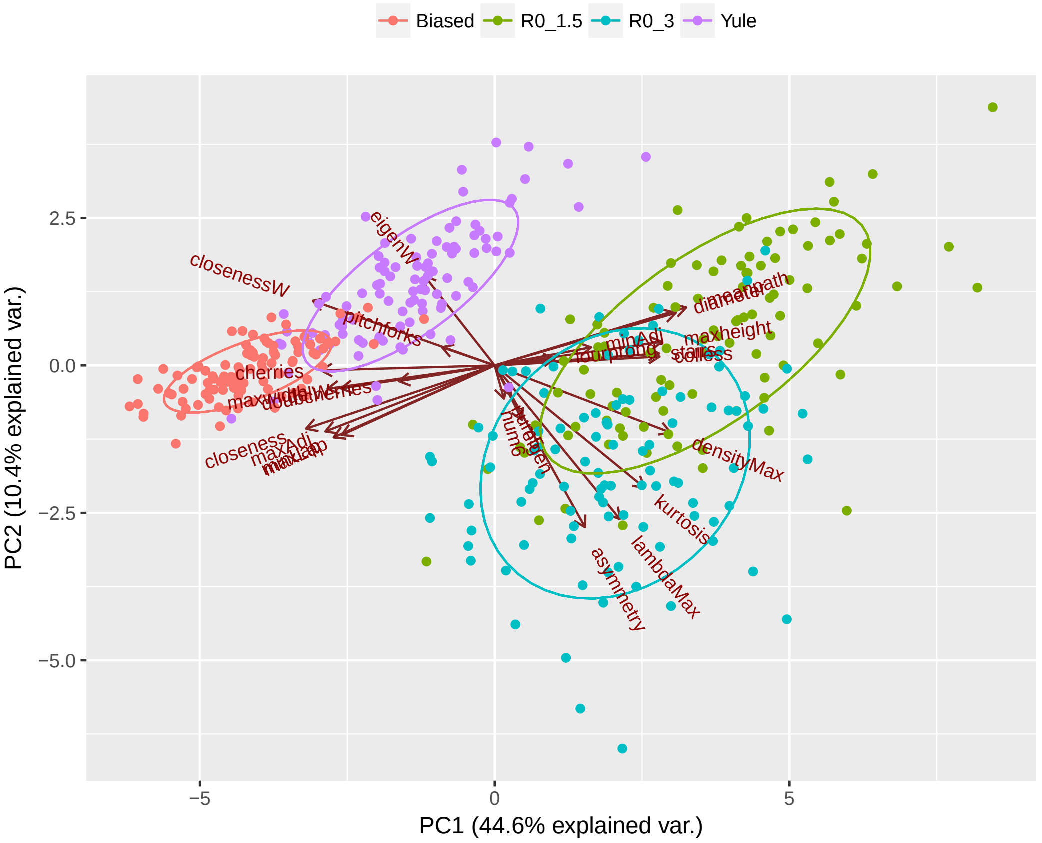

Figure S9: PCA biplots illustrating the separation among phylogenies simulated from a biased model (red), a Yule model (purple) and two birth-death models (green and blue) by using tree shape statistics.

For the uniqueness (in phylogenetic trees), suppose that this there is another node in T_0, say $w \neq u$, that also has the defining property of a centroid. Consider the connected component C^u of T_0 after w is removed that contains u, and the connected component C^w of T_0 after u is removed that contains w. Since u and w are centroids, C^u and C^w each contain fewer than half the nodes, so their union does not contain some node v of T_0. But this cannot happen because if $d(u, v) \leq d(w, v)$ then $v \in C^u$, while if $d(w, v) \leq d(u, v)$ then $v \in C^w$.

Lastly, we note that for any internal node v, there is some tip w such that the path from v to w strictly increases in farness; indeed, it is sufficient to always pick the child of the current node whose subtree contains fewer than half of all the nodes, by equation (1). Therefore, the maximum farness is attained at a tip, not at an internal node.

To connect the concepts of centroid and tricenter, we note that a tricenter, if one exists, is also a centroid, by definition; in particular, a tricenter, if it exists, is always unique; however, a phylogenetic tree always has a centroid, while it may not have a tricenter.

c. Recall the assumption that the tree T has arbitrary positive branch lengths. Let v be a tip with a parent u. The defining equations for the Perron-Frobenius eigenvector specialized to the entries corresponding to u and v say that

$$\lambda e_v = e_u w(uv);$$

$$\lambda e_u = \sum_{w \in \Gamma(u)} e_w w(uw) > e_v w(uv)$$

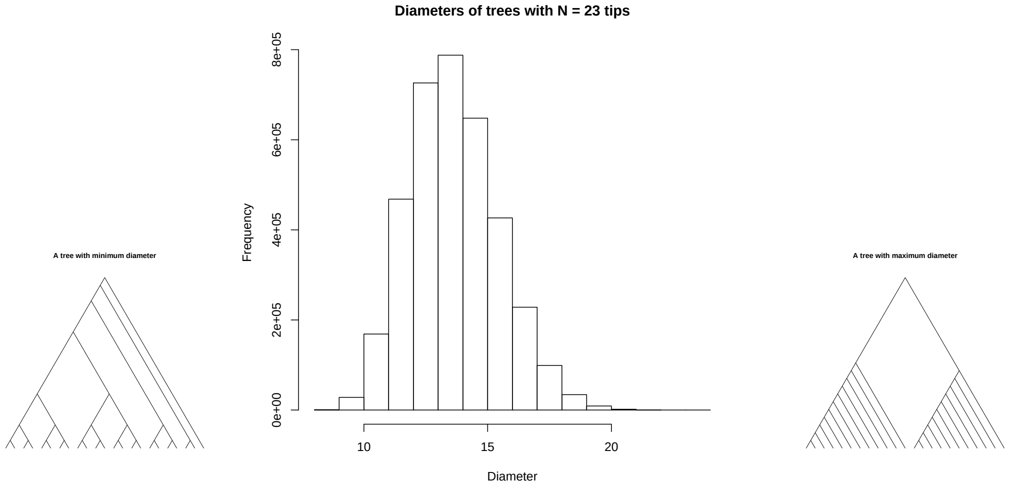

(a) (b) (c)

Figure S10: The distribution of diameters for phylogenetic trees on $n = 23$ tips, with two extremal trees

where the inequality follows from the fact that u has at least one other neighbor, and all the branch lengths and entries of $\vec{e}$ are positive. By cross-multiplying the inequality with the equality, we get

$$\lambda e_u(e_u w(uv)) > e_v w(uv)(\lambda e_v) \implies e_u^2 > e_v^2 \implies e_u > e_v,$$

since $\lambda > 0$ and $w(uv) > 0$. Hence, a tip cannot contain the maximum eigenvector centrality, which is therefore attained at an internal node.

8 The distance Laplacian spectrum does not uniquely define a tree

We give an example of a pair of trees on $n = 3$ tips, with different branch lengths, whose distance Laplacian matrices are co-spectral. We obtained them by exploring all possible integer branch lengths, in order of total branch length (from smallest to largest), and used `Maple` [8] to check, for each resulting weighted topology, the existence of a different set of branch lengths that would result in a distance Laplacian matrix having the exact same characteristic polynomial, and hence the exact same spectrum. The first tree is therefore the tree with integer branch lengths having the smallest possible total length and admitting a co-spectral tree, the second tree, with respect to the distance Laplacian matrix.

The first tree has branch lengths $a = 2$ from the root to the first tip, $b = 2$ from the root to the internal node, and $c = d = 1$ from the internal node to the remaining two tips. The second tree has corresponding branch lengths $a \approx 0.16754417767858494454487$, $b \approx 3.51363991601764694722222$, $c \approx 1.31644667485557576099 55$, $d \approx 0.24554927343936887369 27$ (the exact expressions involve the roots of a polynomial of degree 22). These results were verified using the `RPANDA` package [9] in `R` [10].

9 Correlations between pairs of statistics

We provide an additional figure, Figure S17, that demonstrates the pairwise correlations between all pairs of tree shape statistics considered in our paper, in all five scenarios except for the two

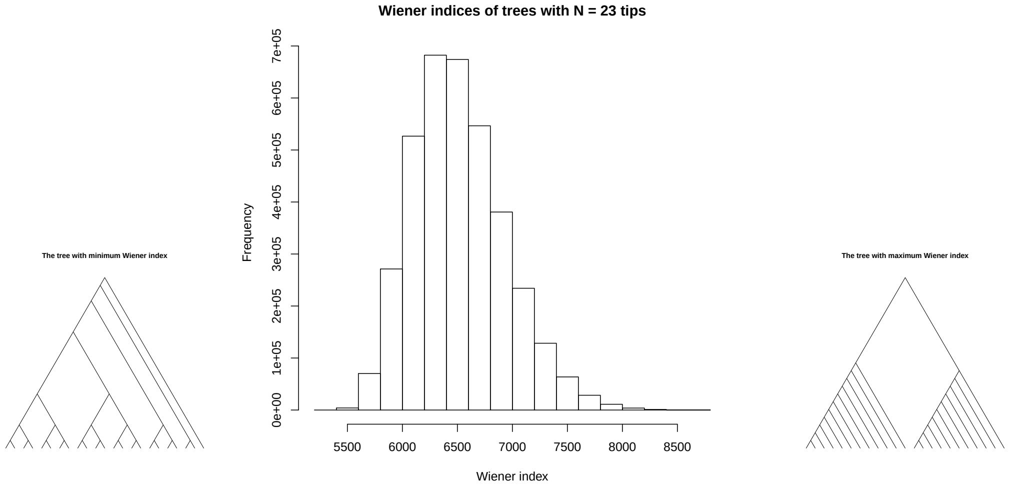

(a) (b) (c)

Figure S11: The distribution of Wiener indices for phylogenetic trees on $n = 23$ tips, with two extremal trees

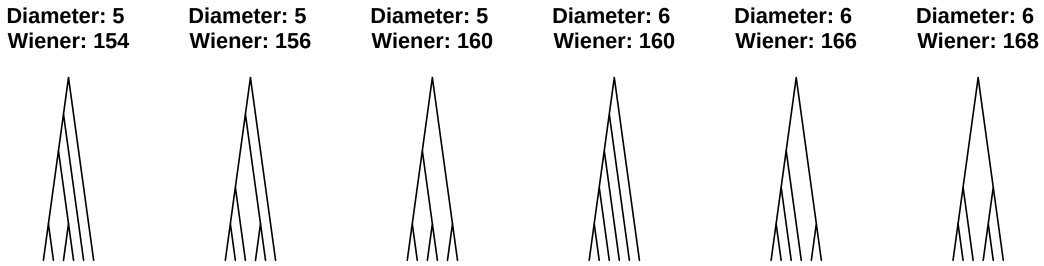

Figure S12: The 6 phylogenetic trees on $n = 6$ tips, with their diameter and Wiener index

simulated ones, which are merged together. These figures suggest general patterns and may be used to help guide the selection process when it is desirable to restrict the number of statistics to be selected. In each case, the darker values indicate larger absolute correlations, with the blue (red) spectrum corresponding to negative (positive) correlations between the statistics.

References

[1] Hillis DM, Heath TA, John KS. Analysis and visualization of tree space. Systematic Biology. 2005;54(3):471–482.

[2] Collins M, Duffy N. Convolution kernels for natural language. In: Advances in Neural Information Processing Systems; 2001. p. 625–632.

[3] Poon AF, Walker LW, Murray H, McCloskey RM, Harrigan PR, Liang RH. Mapping the shapes of phylogenetic trees from human and zoonotic RNA viruses. PLoS One. 2013;8(11):e78122.

[4] Broutin N, Flajolet P. The Distribution of Height and Diameter in Random Non-plane Binary Trees. Random Struct Algorithms. 2012;41(2):215–252.

[5] Entringer RC, Meir A, Moon JW, Székely LA. On the Wiener index of trees from certain families. Australas J Combin. 1994;10:211224.

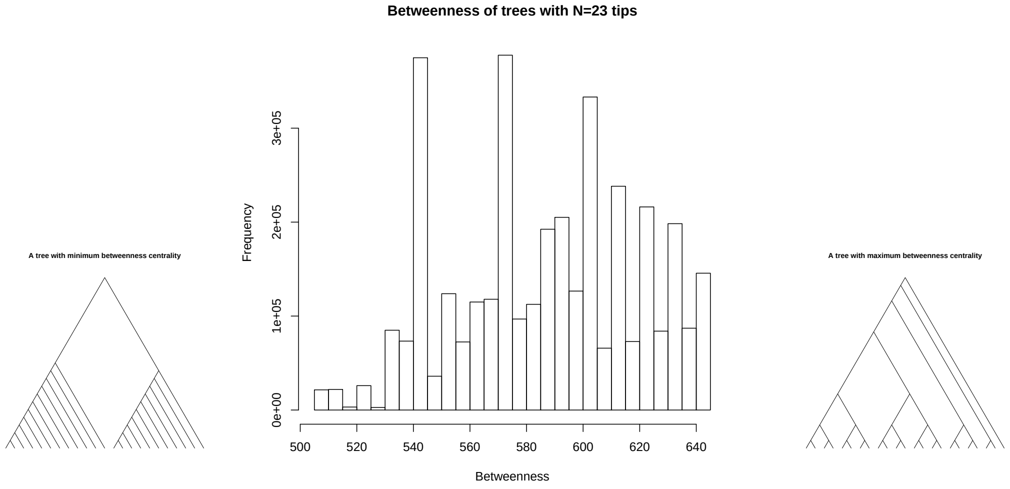

(a) (b) (c)

Figure S13: The distribution of maximum betweenness centrality for phylogenetic trees on $n = 23$ tips, with two extremal trees

[6] Goldman AJ. Optimal Center Location in Simple Networks. Transportation Science. 1971;5(2):212–221.

[7] Jordan C. Sur les assemblages de lignes. Journal für reine und angewandte Mathematik. 1869;70:185–190.

[8] Monagan MB, Geddes KO, Heal KM, Labahn G, Vorkoetter SM, McCarron J, et al. Maple 10 Programming Guide. Waterloo ON, Canada: Maplesoft; 2005.

[9] Morlon H, Condamine F, Lewitus E, Manceau M. RPANDA: an R package for macroevolutionary analyses on phylogenetic trees. Methods in Ecology and Evolution. 2016;R package version 1.0.

[10] R Core Team. R Core Team, editor. R: A Language and Environment for Statistical Computing. Vienna, Austria: R Foundation for Statistical Computing; 2016.

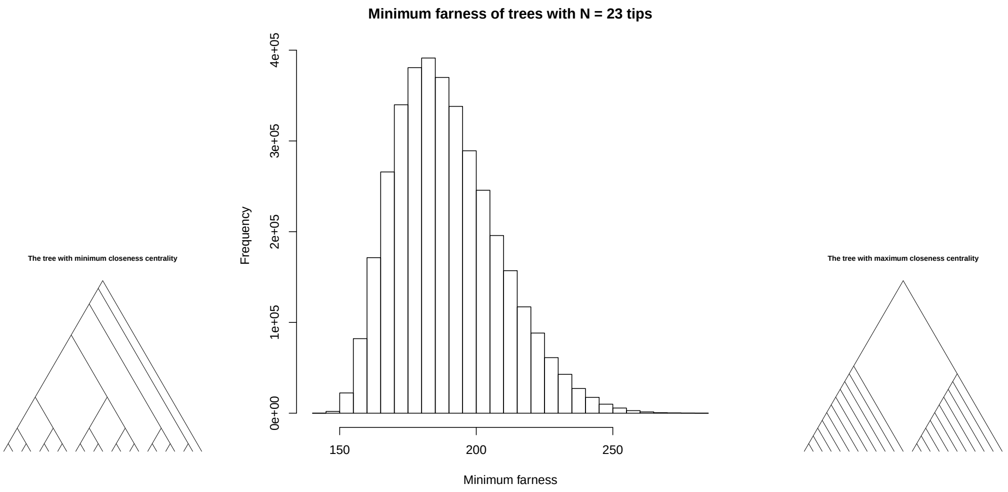

(a) (b) (c)

Figure S14: The distribution of minimum farness (inverse of maximum closeness centrality) for phylogenetic trees on $n = 23$ tips, with two extremal trees

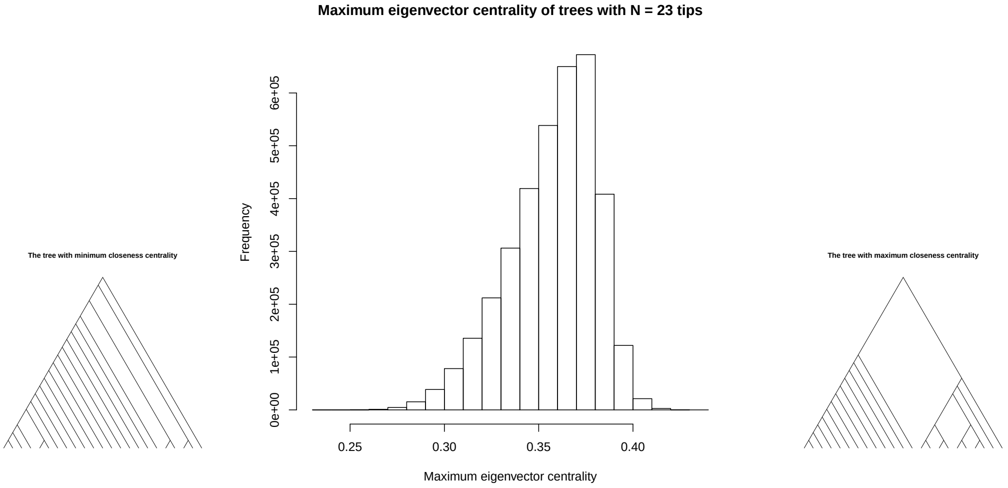

(a) (b) (c)

Figure S15: The distribution of maximum eigenvector centrality for phylogenetic trees on $n = 23$ tips, with two extremal trees

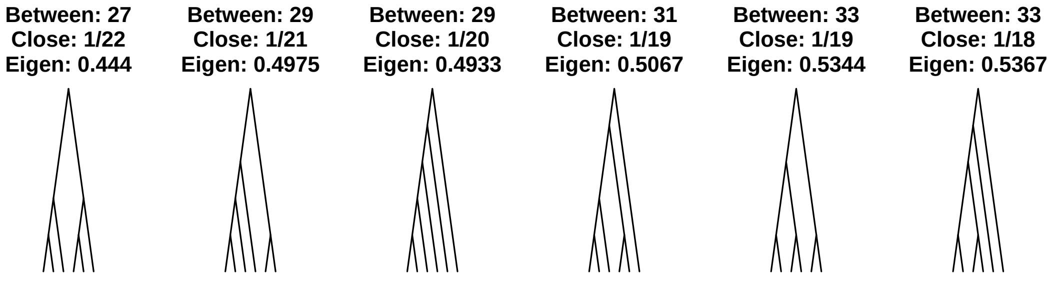

Figure S16: The 6 phylogenetic trees on $n = 6$ tips, with their betweenness, closeness, and eigenvector centrality

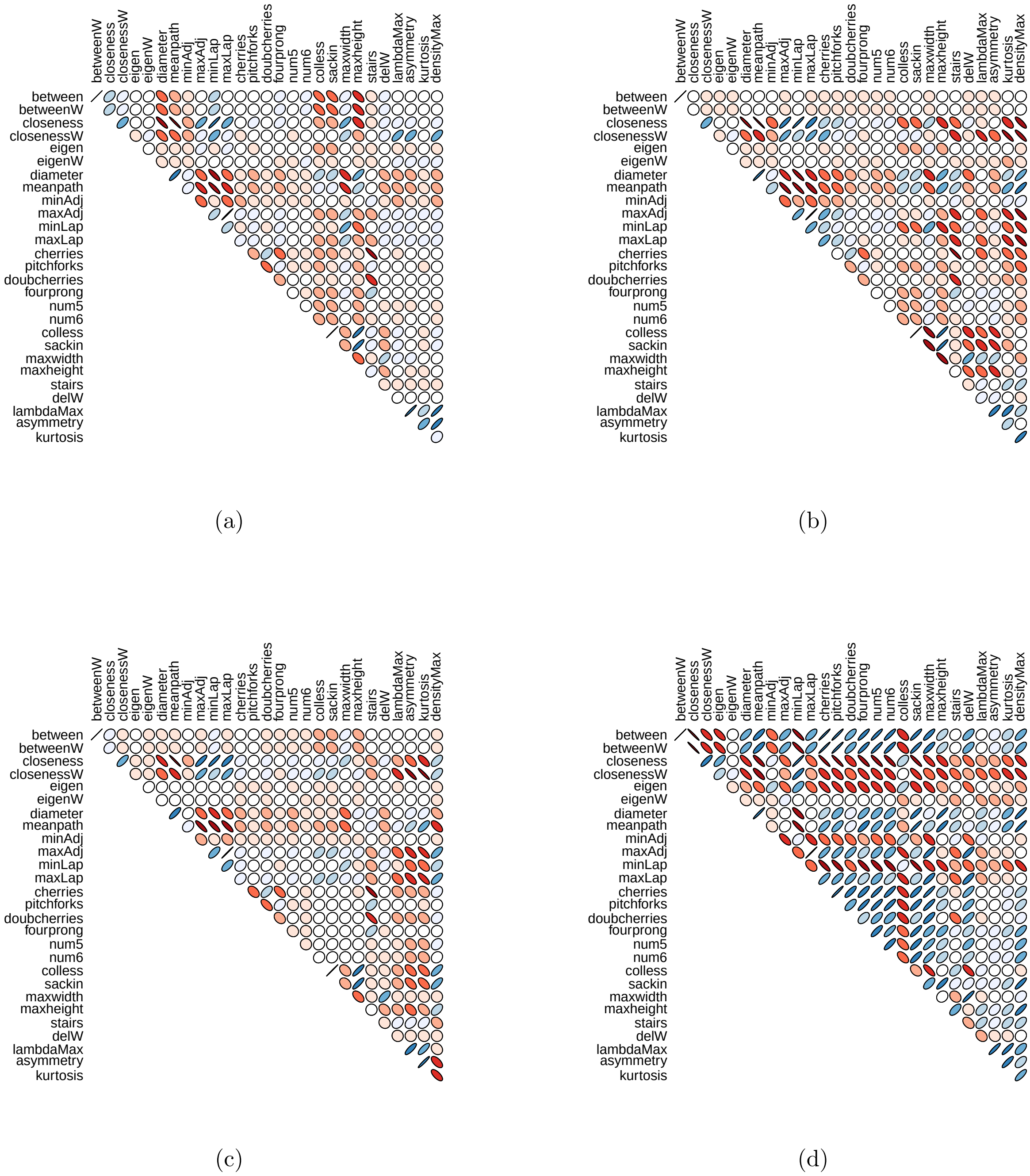

Figure S17: Pairwise correlations between the tree shape statistics in all the scenarios. (a) Flu trees. (b) HIV, dengue and measles trees. (c) HIV trees in three settings. (d) Simulated trees with 100 and 300 tips.
